# Evaluating and selecting arguments in the context of higher order uncertainty

**DOI:** 10.3389/frai.2023.1133998

**Published:** 2023-05-19

**Authors:** Christian Straßer, Lisa Michajlova

**Affiliations:** Institute for Philosophy II, Ruhr University Bochum, Bochum, Germany

**Keywords:** abstract argumentation, probabilistic argumentation, argument strength, higher-order uncertainty, reasoning with uncertainty, non-monotonic logic

## Abstract

Human and artificial reasoning has to deal with uncertain environments. Ideally, probabilistic information is available. However, sometimes probabilistic information may not be precise or it is missing entirely. In such cases we reason with higher-order uncertainty. Formal argumentation is one of the leading formal methods to model defeasible reasoning in artificial intelligence, in particular in the tradition of Dung's abstract argumentation. Also from the perspective of cognition, reasoning has been considered as argumentative and social in nature, for instance by Mercier and Sperber. In this paper we use formal argumentation to provide a framework for reasoning with higher-order uncertainty. Our approach builds strongly on Haenni's system of probabilistic argumentation, but enhances it in several ways. First, we integrate it with deductive argumentation, both in terms of the representation of arguments and attacks, and in terms of utilizing abstract argumentation semantics for selecting some out of a set of possibly conflicting arguments. We show how our system can be adjusted to perform well under the so-called rationality postulates of formal argumentation. Second, we provide several notions of argument strength which are studied both meta-theoretically and empirically. In this way the paper contributes a formal model of reasoning with higher-order uncertainty with possible applications in artificial intelligence and human cognition.

## 1. Introduction

### 1.1. Reasoning with uncertainties

Many sources of information provide uncertain information. Such information may come with probabilistic estimations of how likely specific events are (think of a weather report), in which case we deal with (precise or first order) probabilistic uncertainty. However, often probabilistic information is missing, or the probabilities are not known precisely, in which case we deal with higher-order uncertainty (in short, HOU). HOU occurs when the underlying probability distribution is not or only partially known.^1^ We illustrate the role of HOU with two examples.

**Figure 1:**
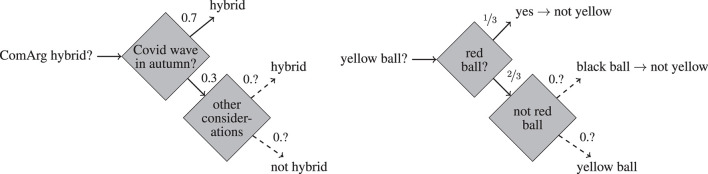
Illustration of Example 1 **(left)** and Example 2 **(right)**. The dashed arrow signify HOU. The numbers on the arrows represent the probabilities of the associated events.

**Example 1** (ComArg). The ComArg conference is to be held during December 2023. We have the following information concerning the question whether ComArg will be held hybrid (see Figure 1, left).

1. The organizers of ComArg announce that a sufficient condition for the conference to be held hybrid is if there is another wave of COVID in autumn.
2. If there is no COVID wave in autumn, the steering committee will take into account other considerations (such as environmental issue, etc.) and decide on this basis whether the conference is to be held hybrid. We lack any information about how likely it is that such considerations lead to a decision in favor (or disfavor) of a hybrid conference.
3. According to expert opinion, the likelihood of a COVID wave in autumn is at 0.7.

The answer to the question whether the next ComArg conference will be held hybrid is uncertain. Moreover, one cannot attach a precise probability to it: the best that can be said is that it has at least the likelihood 0.7 (given statements 1 and 3). We are dealing with HOU in contradistinction to mere first order uncertainty: in contrast to the question how likely a COVID wave in autumn is, the question how likely it is that ComArg will be held hybrid has no precise answer.

**Example 2** (Ellsberg, 1961). Suppose an urn contains 30 red balls, and 60 non-red balls, among which each ball is yellow or black, but we do not know the distribution of yellow and black balls. The question of whether a randomly drawn ball is red is one of first order uncertainty since it comes with the (precise) probability of ⅓. The question whether it is yellow is one of HOU since the available probabilistic information does not lead to a precise probabilistic estimate. See Figure 1 (right) for an illustration.

### 1.2. First and higher-order uncertainty in human cognition and AI (HCAI)

Since our environments come with many sources of uncertain information, both quantifiable and not, it is not surprising that human reasoning is well-adjusted to dealing with such situations. What is more, human reasoning distinguishes the two types of uncertainty by treating them differently. For example, in Example 2 people are more willing to bet on drawing a red ball than on drawing a yellow ball in a game in which one wins if one bets the right color. This phenomenon is known as ambiguity (or uncertainty) aversion. The distinction can be traced back both to the psychological and neurological level. For instance, different types of psychological or other medical problems are associated with a compromised decision making under first order uncertainty, but not under HOU [e.g., gambling problems in Brevers et al. (2012), obsessive-compulsive disorder in Zhang et al. (2015), pathological buying issues in Trotzke et al. (2015)] and vice versa (e.g., Parkinson's disease in Euteneuer et al., 2009). This shows that different causal mechanisms are related to the human capacities of reasoning with the two types of uncertainties. Similarly, on the neurological level differences can be traced, though it is still an open issue whether the two uncertainty types have separate or graded representations in the brain [see De Groot and Thurik (2018), to which we also refer for a recent overview on both the psychological and neurological literature].

What the discussion highlights is that a formal model of human reasoning should pay special attention to both types of uncertainties and provide a framework that can integrate mixed reasoning processes, such as in our Examples 1 and 2. The same can be stated for AI for the simple reason that in many applications artificial agents will face sources of uncertain information.

When reasoning with uncertain information, we infer defeasibly, that is, given new (and possibly more reliable) information we may be willing to retract inferences. As forcefully argued on philosophical grounds in Toulmin (1958), reasoning is naturally studied as a form of argumentation. Similarly, the cognitive scientists Mercier and Sperber developed an argumentative theory of human reasoning (Mercier and Sperber, 2017). Dung's abstract argumentation theory (Dung, 1995) provides a unifying formal framework for an argumentative model of defeasible reasoning and has been widely adopted by now both in the context of symbolic AI and to provide explanatory frameworks in the context of human cognition (Saldanha and Kakas, 2019; Cramer and Dietz Saldanha, 2020). Several ways of instantiating abstract argumentation with concrete formal languages and rule sets have been proposed, such as ASPIC+ (Modgil and Prakken, 2014), assumption-based argumentation (Dung et al., 2009), and logic-based argumentation (Besnard and Hunter, 2001; Arieli and Straßer, 2015).

It would therefore seem advantageous for the theoretical foundations of HCAI to combine formal argumentation with a representation of first and higher-order uncertainty. This paper will propose such a formal framework.

### 1.3. Formal methods

Several formal models of this type of reasoning are available: from imprecise probabilities (Bradley, 2019) to subjective logic (Jøsang, 2001) and probabilistic argumentation (Haenni, 2009). However, the link to the leading paradigm of computational argumentation, namely Dung-style argumentation semantics, is rather loose.

Probabilistic argumentation with uncertain probabilities is comparatively understudied in formal argumentation. Works by Hunter and Thimm (Hunter and Thimm, 2017; Hunter, 2022) focus on precise probabilities. Our framework generalizes aspects of such settings to include a treatment of HOU. Also, in contrast to them, we will utilize Dung argumentation semantics in the context of probabilistic argumentation. Hunter et al. (2020) equip arguments with a degree of belief as well as disbelief, notions that can also be expressed in Haenni's framework and will be considered in our study of argument strength. A framework that considers imprecise probabilities is presented by Oren et al. (2007). It utilizes subjective logic in the context of a dialogical approach for reasoning about evidence. Similarly, Santini et al. (2018) label arguments in abstract argumentation with opinions from subjective logic. In contrast, our study focuses on structured argumentation.

Mainly starting with the seminal (Ellsberg, 1961), HOU has been intensively studied in the context of decision theory. As has been shown there, human reasoning with HOU may lead to violations of axioms of subjective expected utility theory (as axiomatized in Savage, 1972), leading to several alternative accounts [e.g., maxmin expected utility in Gilboa and Schmeidler (2004) or prospect theory in Kahneman and Tversky (1979)]. In this paper, we omit utilities, values, and practical decision making and concentrate instead on reasoning in the epistemic context of belief formation and hypothesis generation. As we will show, even without utilities HOU gives rise to interesting and challenging reasoning scenarios.

### 1.4. Our contribution

In this paper we integrate reasoning with HOU in abstract argumentation. For achieving this goal, several key questions have to be answered:

1. *What is a knowledge base?* A knowledge base contains strict assumptions (also, constraints) and defeasible assumptions for which probabilistic information is available in form of a family of probability functions. Following Haenni (2009), we distinguish probabilistic and non-probabilistic (also, logical) variables, where only for the former set probabilistic information is available.
2. *What is a logically structured argument?* We will follow the tradition in logical/deductive argumentation according to which an argument is a pair 〈S,ϕ〉 where S is a set of assumptions and ϕ a sentence that deductively follows from S (in signs, S⊢ϕ).
3. *When is an argument stronger than another one?* We propose several measures of argument strength with special consideration of HOU and study their properties. Any model of defeasible reasoning may have various applications, from normative philosophical models of non-monotonic inference to symbolic artificial intelligence, to descriptively adequate (and therefore predictive) models of human reasoning. When considering argument strength, we here focus on the latter and provide a small empirical study (incl. well-known reasoning tasks such as Ellsberg, 1961) to check the accuracy of the previously defined notions. Clearly, this is a first preliminary step which can only point in a direction, rather than conclusively validate the formal notions developed in this paper.^2^
4. *What constitutes an argumentative attack?* We study four types of argumentative attack, namely, rebut and three forms of undercut.
5. *How to obtain meta-theoretically well-behaved selections of arguments?* We study several standard argumentation semantics from Dung (1995) for different attack forms in terms of rationality postulates developed for structured argumentation Caminada and Amgoud (2007). When applying argumentation semantics, problems concerning the consistency of extensions already known from logical argumentation re-occur: namely, the set of conclusions of arguments in a given complete extension may be inconsistent. We will propose a solution to this problem that is also applicable in the context of probabilistic argumentation in the style of Hunter and Thimm (2017) and logical argumentation. Moreover, we argue that a naive selection of arguments whose strength passes a certain threshold can lead to inconsistency. Instead, selections in the tradition of Dung seem to be more promising. First, our Dung-based approach satisfies several rationality postulates (including some concerning the consistency of selections). Moreover, it allows for the reinstatement of arguments that are defended by other selected arguments from attacks by non-selected arguments. This is advantageous e.g., when adopting an investigative or hypothetical reasoning style.

Our work takes as the starting point the theory of probabilistic argumentation developed in Haenni (2009). The framework is enhanced by (1) a structured notion of argument in the style of logical argumentation, (2) argumentative attacks, (3) several notions of argument strength [based on notions of degree of support and degree of possibility presented in Haenni (2009)], and (4) a study of Dung-style argumentation semantics. This way, we obtain a generalization of both (some forms of) logical argumentation (Besnard and Hunter, 2018) and probabilistic argumentation in the tradition of Hunter and Thimm (2017).

The structure of the paper is as follows. In Section 2, we introduce knowledge bases and arguments. In Section 3, we discuss the application of argumentation semantics and study rationality postulates relative to the attack form used. Section 4 presents the empirical study on argument strength. We provide a discussion and conclusion in Section 5. In the Appendix (Supplementary material), we provide proofs of our main results, some alternative but equivalent definitions, and details on our empirical study.

## 2. Knowledge bases and arguments

### 2.1. Knowledge bases

Our reasoning processes never start from void, but we make use of available information when building arguments. This available information is encoded in a knowledge base. In our initial Example 1, we had two types of information available:

1. probabilistic information concerning a COVID-wave (“the likelihood of a COVID wave in autumn is 70%”). This information may ground defeasible assumption such as “a COVID wave will (probably) (not) take place”; and
2. information about a factual constraint concerning the circumstances in which the conference will be held hybrid (namely, if there is a COVID wave in autumn).

More generally we will follow this rough distinction in probabilistic information that gives rise to defeasible assumptions, on the one hand, and factual constraints, on the other hand. Constraints are taken for granted, either because a reasoner is convinced of their truth, or otherwise committed to them in the reasoning process (e.g., they may be supposed in an episode of hypothetical reasoning^3^).

Altogether a knowledge base consists of the following components:

**Assumptions**. Our knowledge bases are equipped with a (finite) set of propositional variables $V_{p}$ about which probabilistic information (in the form of probability functions) is available. Out of these propositional variables a set of *defeasible assumptions* A is formed, whose strength will be influenced by their probabilities. So, where $\text{sent}\left(V_{p}\right)$ is the set of the propositional sentences with atoms in $V_{p}$, $A\subseteq \text{sent}\left(V_{p}\right)$.
**Probabilities**. We work with a *set probability functions* ℙ based on $\text{states}\left(V_{p}\right)$, where $\text{states}\left(V_{p}\right)$ is the state space for $V_{p}$ (i.e., the set of all truth-value assignments $v:V_{p}\to \left\{0,1\right\}$). This allows for cases in which more than one probability function is available, e.g., scenarios in which multiple sources of probabilities are considered or in which the probabilistic information about the state space $\text{states}\left(V_{p}\right)$ is incomplete (see below for examples).

For a sentence ϕ we let ∥ϕ∥ be the set of states $s\in \text{states}\left(V_{p}\right)$ for which *s*⊨ϕ. For some $s\in \text{states}\left(V_{p}\right)$, we denote by ŝ the conjunction $\wedge \left\{\phi |\alpha \left(\phi \right)=1,\phi \in V_{p}\right\}\cup \left\{\neg \phi |\alpha \left(\phi \right)=0,\phi \in V_{p}\right\}$. The reader finds an overview of the notation used in this paper in Table 1.

**Table 1:** Overview: technical notation.

**Syntactic entities**	
*p, q*, …	Propositional atoms
ϕ, ψ, …	Propositional sentences
Γ	Set of sentences
ŝ	Syntactic representation of state *s*
$V_{p}$	Set of probabilistic propositional variables
$V_{l}$	Set of logical propositional variables
sent(V)	Set of sentences over V
A	Set of assumptions (subset of $\text{sent}\left(V_{p}\right)$)
C	Set of constraints (subset of $\text{sent}\left(V_{p}\cup V_{l}\right)$)
𝕂	Knowledge base
*a, b*, …	Arguments
Sup(*a*)	Support of *a*
Con(*a*)	Conclusion of *a*
Arg(𝕂)	Set of arguments induced by 𝕂
@(Γ)	The argument 〈Γ, ∧Γ〉
E	Set of arguments, an argumentation extension
**Semantic entities**	
*s*	State
states(V)	Set of states over variables in V
*P*	Probability function
$P^{C}$	Probability function after Bayesian update on C
ℙ	Set of probability functions
℘(·)	Power set
∥ϕ∥	Set of states that verify ϕ
$∥\phi {∥}_{C}$	Set of states that verify ϕ and are consistent with C
str(*a*)	Placeholder for argument strength function
dsp(*a*)	Degree of support of *a*
dps(*a*)	Degree of possibility of *a*

We may *also* use propositional variables for which no (direct) probabilistic information is considered. We collect these in $V_{l}$ (the logical variables) and require $V_{l}\cap V_{p}=\emptyset$. By allowing logical variables as well as probabilistic ones, we can unite logical (where $V_{p}=\emptyset$) and probabilistic reasoning (where $V_{l}=\emptyset$) and can involve both systems seamlessly, following the approach by Haenni (2009). Constraints will typically relate probabilistic with non-probabilistic information and therefore they are based on atoms from both sets, $V_{p}$ and $V_{l}$.

**Constraints**. The last element of knowledge bases is a set of factual constraints (in short, *constraints*) C. The formulas in C are based on atoms in $V_{p}\cup V_{l}$.

In the following we write $\Gamma {⊢}_{C}\phi$ as an abbreviation of Γ∪C⊢ϕ, where $\Gamma \subseteq \text{sent}\left(V_{l}\cup V_{p}\right)$. It will also be useful to collect all states that are consistent with C and that support a formula $\phi \in \text{sent}\left(V_{l}\cup V_{p}\right)$ in ${∥\phi ∥}_{C}=\left\{s\in \text{states}\left(V_{p}\right)|ŝ{⊬}_{C}\neg \phi \text{and}ŝ{⊢}_{C}\phi \right\}$. Similarly, we write $s{⊨}_{C}\phi$ in case $s\in {∥\phi ∥}_{C}$.

We summarize the above discussion in the following definition:

**Definition 1** (Knowledge Base). A knowledge base 𝕂 is a tuple $\langle \langle V_{p},V_{l}\rangle ,A,C,\mathbb{P}\rangle$ for which

- $V_{p}$ is a finite set of *probabilistic variables*,
- $V_{l}$ is a finite set of *logical variables* such that $V_{l}\cap V_{p}=\emptyset$,^4^
- $A\subseteq \text{sent}\left(V_{p}\right)$ is a finite set of *defeasible assumptions*,
- $C\subseteq \text{sent}\left(V_{p}\cup V_{l}\right)$ is a finite set of *constraints*,
- ℙ is a non-empty set of probability functions $P:\text{states}\left(V_{p}\right)\to \left[0,1\right]$.

**Example 3** (Ex. 1 cont.). Let us return to our example. It can be modeled by the knowledge base


$$\mathbb{C}𝕆𝕍𝕀𝔻=\langle \langle V_{p}:\left\{p\right\},V_{l}:\left\{q\right\}\rangle ,A:\left\{p,\neg p\right\},C:\left\{p\to q\right\},\mathbb{P}:\left\{P\right\}\rangle ,$$

where *P*(*p*) = 0.7, *p* stands for a COVID-Wave to happen and *q* for the conference to by held hybrid. Our defeasible assumptions A are {*p*, ¬*p*} (“there will (not) be a COVID-Wave”). Our set of factual constraints is C={p→q}. Table 2 shows the state space induced by $V_{p}$.

**Table 2:** The state space and the probabilities for Example 3.

**State**	***s* ⊨ *p***	***P***
*s*_1_		0.3
*s*_2_	✓	0.7

### 2.2. Arguments, support, and strength

Given a knowledge base 𝕂=〈A,C,ℙ〉, a natural way of thinking about arguments is in terms of support-conclusion pairs:

**Definition 2** (Argument). Given a knowledge base 𝕂=〈A,C,ℙ〉, an *argument* *a* for 𝕂 is a pair 〈Sup(*a*), Con(*a*)〉, where

- Sup(a)⊆A is a set of assumptions, the *support* of *a*,
- $Con\left(a\right)\in \text{sent}\left(V_{l}\cup V_{p}\right)$ is the *conclusion* of *a*,
- such that $Sup\left(a\right){⊢}_{C}Con\left(a\right)$.

We write Arg(𝕂) for the set of all arguments based on 𝕂.

**Example 4** (Ex. 3 cont.). In our example we can form the argument hybrid = 〈{*p*}, *q*〉 for the conference to be held hybrid, the argument wave = 〈{*p*}, *p*〉 for there being a COVID-wave, and noWave = 〈{¬*p*}, ¬*p*〉 for there being no wave.

When considering the question of how strong an argument *a* = 〈Γ, ϕ〉 is, a naive approach is to simply measure the probabilistic strength of the support. In the simple case of our example and the argument hybrid = 〈{*p*}, *q*〉 this would amount to *P*(*p*) = 0.7, the same as for the argument wave, whereas noWave would only have a strength of 0.3. However, there are some subtleties which motivate a more fine-grained analysis. To show this, we enhance our example as follows.

**Example 5** (ComArg2). We also consider another conference, ComArg2, for which we know that it will be held hybrid (symbolized by *q*′) if and only if(!) a COVID-wave breaks in autumn. Our enhanced knowledge base is ${\mathbb{C}𝕆𝕍𝕀𝔻}^{\prime }=\langle \langle \left\{p\right\},\left\{q,q^{\prime }\right\}\rangle ,A:\left\{p,\neg p\right\},C^{\prime }:\left\{p\to q,p↔q^{\prime }\right\}\rangle$. We now added also *p* ↔ *q*′ to the set of constraints C. We can now also consider the additional argument hybrid′ = 〈{*p*}, *q*′〉 for ComArg2 to be held hybrid.

**Observation 1** (Stronger, but less precise arguments.). Intuitively, the argument hybrid in favor of *q* is stronger than the argument hybrid′ in favor of *q*′ (see also our empirical study in Section 4). Although both arguments have the same support, {*p*}, the conclusion *q* of hybrid is compatible with both states, *s*_1_ and *s*_2_ (it is certain in *s*_2_ and possible in *s*_1_), while the conclusion *q*′ of hybrid′ is only compatible with *s*_2_. As a consequence, *q* has *at least* the probability 0.7, while *q*′ has *precisely* the probability 0.7.

In sum, it is intuitive to consider an argument *a* as at least as strong as an argument *b*, in case both arguments have the same support, but the conclusion of *a* is at least as probable as the conclusion of *b*.

Let us analyse this observation in more formal terms. We write $s{⊨}_{C}◇\phi$ iff $s\in ∥⊤{∥}_{C}\backslash ∥\neg \phi {∥}_{C}$. This means that ϕ is possible in *s* in view of the constraints in C. Similarly, we write $∥◇\phi {∥}_{C}$ for the set of states $∥⊤{∥}_{C}\backslash ∥\neg \phi {∥}_{C}$.

**Fact 1**. Let *a* ∈ Arg(𝕂) and $Con\left(a\right){⊢}_{C}\phi$. Then (1) $∥Sup\left(a\right){∥}_{C}=∥\wedge Sup\left(a\right){∥}_{C}\subseteq ∥Con\left(a\right){∥}_{C}\subseteq ∥\phi {∥}_{C}$, and (2) $∥Con\left(a\right){∥}_{C}\subseteq ∥◇Con\left(a\right){∥}_{C}\subseteq ∥◇\phi {∥}_{C}$.

In our Example 5 we have the validities for the different states shown in Table 3. Following Observation 1, hybrid = 〈{*p*}, *q*〉 is stronger than hybrid′ = 〈{*p*}, *q*′〉. The reason seems to be that despite having the same support, the “space of possibility” for *q* is larger than the one for *q*′: {*s*_1_, *s*_2_} vs. {*s*_2_}. From the probabilistic perspective, the support for *p* seems to be located in [0.7, 1] while the one for *q*′ is exactly 0.7.

**Table 3:** Validities for Example 5.

**State *s***	***s* ⊨ *p***	** $s{\;⊨\;}_{C^{\prime }}\;q$ **	** $s{\;⊨}_{C^{\prime }}\;◇q$ **	** $s{\;⊨}_{C^{\prime }}\;q^{\prime }$ **	** $s{\;⊨}_{C^{\prime }}\;◇q^{\prime }$ **
*s*_1_			✓		
*s*_2_	✓	✓	✓	✓	✓

Following this rationale, the strength of the support of an argument is measured relative to a lower and upper bound: the lower bound is the cautious measure of how probable the support is in the *worst case*, the upper bound considers the *best case* scenario in which states in which the conclusion holds have maximal probability mass. As we will see, the central idea for modeling argument strength in this paper is by means of functions that map arguments to [0, 1] (their strength) by aggregating the worst and best case support.

Before discussing two complications, we shortly summarize the ideas so far. Arguments are support-conclusion pairs. When considering the strength of an argument *a* = 〈Sup, ϕ〉 it is advisable not only to consider the probabilistic strength of its support Sup, but also to consider the probabilistic support for the possibility of its conclusion ◇ϕ. A measure of argument strength is expected to aggregate the two.

### 2.3. Imprecise probabilistic information

In many scenarios it will be advantageous or unavoidable to work with families of probability functions, instead of a unique probability function. These are cases in which the probabilistic information concerning the probabilistic variables in $V_{p}$ is incomplete or it stems from various sources, each providing an individual probability function. The following example falls in the former category.

**Example 6** (Three conferences). Peter and Mary are in the steering committee of ConfB, ConfP, and ConfM. Their votes have different weights for the decision making of the respective committees. Both of their positive votes are sufficient but not necessary for ConfB to be held hybrid. For ConfP the decision relies entirely on Peter's vote, and for ConfM it relies entirely on Mary's vote.

- If Peter votes hybrid, ConfB will be hybrid.      *p*_1_ → *q*_1_
- If Mary votes hybrid, ConfB will be hybrid.      *p*_2_ → *q*_1_
- ConfP will be hybrid if and only if Peter votes hybrid.      *p*_1_ ↔ *q*_2_.
- ConfM will be hybrid if and only if Mary votes hybrid.      *p*_2_ ↔ *q*_3_.
- According to Peter, there is a ⅔ likelihood that he will vote hybrid.      *P*(*p*_1_) = ⅔
- According to Mary, she is at least as likely to vote hybrid as Peter.      *P*(*p*_2_) ≥ ⅔
  (We lack more precise information.)

Altogether our knowledge base is given by $𝕂=\langle \langle \left\{p_{1},p_{2}\right\},\left\{q_{1},q_{2},q_{3}\right\}\rangle ,A,C,\mathbb{P}:\left\{P_{\mu }|\mu \in \left[0,⅓\right]\right\}\rangle$, where $A=\text{sent}\left(V_{p}\right)$ and $C=\left\{p_{1}\to q_{1},p_{2}\to q_{1},p_{1}↔q_{2},p_{2}↔q_{3}\right\}$. Moreover, in this case the probabilities for our defeasible assumptions *p*_1_ and *p*_2_ are not precise. They are expressed by means of a family of probability functions (see Table 4).^5^

**Table 4:** The state space and probabilities for Example 6, where μ ∈ [0, ⅓].

**State**	***p*_1_**	***p*_2_**	***P*_μ_**	***P*_μ = 0_**	***P*_μ = ⅓_**	***q*_1_**	***q*_2_**	***q*_3_**
*s*_1_	0	0	⅓ · (⅓ − μ)	⅑	0	◇	0	0
*s*_2_	0	1	⅓ · (⅔+μ)	^2^/_9_	⅓	1	0	1
*s*_3_	1	0	⅔ · (⅓ − μ)	^2^/_9_	0	1	1	0
*s*_4_	1	1	⅔ · (⅔ + μ)	^4^/_9_	⅔	1	1	1

Given an argument 〈Sup, ϕ〉, a cautious way to consider the worst case probabilistic support is by considering $\inf_{P\in \mathbb{P}}\left(P\left(∥Sup{∥}_{C}\right)\right)$. Following Haenni, we refer to this measure as the *degree of support* of an argument. For the best case probabilistic support, on the other hand, we consider $\sup_{P\in \mathbb{P}}\left(P\left(∥◇\phi {∥}_{C}\right)\right)$. We refer to this measure as the *degree of possibility* of an argument. An overview for the current example can be found in Table 5. Before formally defining the two discussed measures, we have to still consider one more complication, however, which will discuss in the next section.

**Table 5:** The degrees of support and possibility for Example 6.

**Argument**	**Degree of support**	**Degree of possibility**	**Precision**	**Imprecision**
*a*_1_ = 〈*p*_1_, *q*_1_〉	$\begin{array}{c} \inf_{\mu \in \left[0,⅓\right]}\left(P_{\mu }\left(∥p_{1}∥\right)\right)=\\ \inf\left(\left\{⅔\right\}\right)=⅔ \end{array}$	$\begin{array}{c} \sup_{\mu \in \left[0,⅓\right]}\left(P_{\mu }\left(∥◇q_{1}∥\right)\right)=\\ \sup\left(\left\{1\right\}\right)=1 \end{array}$	⅔	⅓
*a*_2_ = 〈*p*_2_, *q*_1_〉	$\begin{array}{c} \inf_{\mu \in \left[0,⅓\right]}\left(P_{\mu }\left(∥p_{2}∥\right)\right)=\\ \inf\left(\left[⅔,1\right]\right)=⅔ \end{array}$	$\begin{array}{c} \sup_{\mu \in \left[0,⅓\right]}\left(P_{\mu }\left(∥◇q_{1}\right)∥\right)=\\ \sup\left(\left\{1\right\}\right)=1 \end{array}$	⅔	⅓
*a*_3_ = 〈*p*_1_∨*p*_2_, *q*_1_〉	infμ∈[0,⅓](Pμ(∥p1∨p2∥))=inf([89,1])=89	$\begin{array}{c} \sup_{\mu \in \left[0,⅓\right]}\left(P_{\mu }\left(∥◇q_{1}\right)∥\right)=\\ \sup\left(\left\{1\right\}\right)=1 \end{array}$	^8^/_9_	⅑
*b* = 〈*p*_1_, *q*_2_〉	$\begin{array}{c} \inf_{\mu \in \left[0,⅓\right]}\left(P_{\mu }\left(∥p_{1}∥\right)\right)=\\ \inf\left(\left\{⅔\right\}\right)=⅔ \end{array}$	$\begin{array}{c} \sup_{\mu \in \left[0,⅓\right]}\left(P_{\mu }\left(∥◇q_{2}∥\right)\right)=\\ \sup\left(\left\{⅔\right\}\right)=⅔ \end{array}$	1	0
*c* = 〈*p*_2_, *q*_3_〉	$\begin{array}{c} \inf_{\mu \in \left[0,⅓\right]}\left(P_{\mu }\left(∥p_{2}∥\right)\right)=\\ \inf\left(\left[⅔,1\right]\right)=⅔ \end{array}$	$\begin{array}{c} \sup_{\mu \in \left[0,⅓\right]}\left(P_{\mu }\left(∥◇q_{3}∥\right)\right)=\\ \sup\left(\left[⅔,1\right]\right)=1 \end{array}$	⅔	⅓

### 2.4. Updating the probabilities in view of the constraints

Consider the following example:

**Example 7** (Witnesses). 1. According to witness 1 *p* ∧ *q* is the case.      *p*_1_ → *p* ∧ *q*

2. According to witness 2 *p* ∧ ¬*q* is the case.      *p*_2_ → *p* ∧ ¬*q*
3. Witness 1 tells the truth in ⅔ of cases.      *P*(*p*_1_) = ⅔
4. Witness 2 tells the truth in ¾ of cases.      *P*(*p*_2_) = ¾

We may model this scenario with the knowledge base $𝕂=\langle \langle V_{p}:\left\{p_{1},p_{2}\right\},V_{l}:\left\{p,q\right\}\rangle ,A:\text{sent}\left(V_{p}\right),C:\left\{p_{1}\to p\wedge q,p_{2}\to p\wedge \neg q\right\},\mathbb{P}=\left\{P\right\}\rangle$ where *P* assigns the probabilities as depicted in Table 6.

**Table 6:** The states for Example 7.

**State *s***	***p*_1_**	***p*_2_**	***P***	** $s\in ∥⊤{∥}_{C}$ **	** $P^{C}\left(s\right)$ **	** $s{⊨}_{C}p$ **	** $s{⊨}_{C}◇p$ **
*s*_1_	0	0	^1^/_12_	✓	^*P*(*s*_1_)^/_*P*({*s*_1_, *s*_2_, *s*_3_})_ = ⅙		✓
*s*_2_	0	1	¼	✓	^*P*(*s*_2_)^/_*P*({*s*_1_, *s*_2_, *s*_3_})_ = ½	✓	✓
*s*_3_	1	0	⅙	✓	^*P*(*s*_3_)^/_*P*({*s*_1_, *s*_2_, *s*_3_})_ = ⅓	✓	✓
*s*_4_	1	1	½		^*P*(∅)^/_*P*({*s*_1_, *s*_2_, *s*_3_})_ = 0		

The 5th column indicates which states are consistent with C (the only exception is state *s*_4_). The 6th column represents the updated probabilities for each state in accordance with Equation (1).

In this case *s*_4_ is incompatible with the set of constraints C of our knowledge in 𝕂 and the probabilities have to be updated. We follow Haenni (2009) by using a Bayesian update on ∧C and letting


(1) $$\begin{array}{c} P^{C}\left(s\right)=\frac{P\left(s\right)}{P\left(∥C∥\right)}\cdot P\left(∥C∥|s\right)=\frac{P\left(s\right)}{P\left(∥⊤{∥}_{C}\right)}\cdot \frac{P\left(∥s{∥}_{C}\right)}{P\left(s\right)}=\frac{P\left(∥ŝ{∥}_{C}\right)}{P\left(∥⊤{∥}_{C}\right)}. \end{array}$$

Similarly, where ℙ is a family of probability functions, we let ${\mathbb{P}}^{C}=\left\{P^{C}|P\in \mathbb{P}\right\}$. When calculating the degrees of support and degrees of possibility of an argument we will consider ${\mathbb{P}}^{C}$ instead of ℙ.

**Definition 3** (Degree of Support and Degree of Possibility, (Im)Precision). Given a knowledge base 𝕂=〈A,C,ℙ〉 and an argument *a* = 〈Sup, ϕ〉 for 𝕂,

- The degree of support of *a* (in signs, dsp(*a*)) is given by $\inf_{P^{C}\in {\mathbb{P}}^{C}}\left(P^{C}\left(∥Sup∥\right)\right)$,
- The degree of possibility of *a* (in signs, dps(*a*)) is given by $\sup_{P^{C}\in {\mathbb{P}}^{C}}\left(P^{C}\left(∥◇\phi ∥\right)\right)$,
- The imprecision of *a* (in signs, imprec(*a*)) is given by dps(*a*)−dsp(*a*),
- The precision of *a* (in signs, prec(*a*)) is given by 1−imprec(*a*).

**Fact 2**. Let *a, b* ∈ Arg(𝕂).

1. If Sup(*a*) ⊆ Sup(*b*) then dsp(*a*) ≥ dsp(*b*).
2. If $\left\{Con\left(a\right)\right\}{⊢}_{C}Con\left(b\right)$ then dps(*b*) ≥ dps(*a*).

As discussed above, we expect a measure of argument strength to aggregate the two measures of degree of support and degree of possibility.

**Definition 4** (Argument strength function). Let 𝕂=〈A,C,ℙ〉 be a knowledge base. A measure of argument strength for 𝕂 is a function str:Args(𝕂) → [0, 1] that is associated with a function π : Θ → [0, 1] for which Θ = {(*n, m*) ∈ [0, 1]^2^ ∣*n* ≤ *m*} and str(*a*) = π(dsp(*a*), dps(*a*)).

## 3. Argument selection

In this section, we consider the question of how to evaluate the strength of arguments and how to select them for acceptance out of a scenario of possibly conflicting arguments. The questions of argument strength and of argument selection are connected: e.g., if two arguments conflict, it is usually advisable to select the stronger of the two. We will proceed in several steps.

1. We propose several notions of argument strength and study their properties (Section 3.1).
2. In Section 3.2, we discuss two types of argumentative attacks: rebuttals and undercuts. We show that both lead to suboptimal outcomes when combined with Dung-style argumentation semantics for selecting arguments in a naive way.
3. In Section 3.3, we propose a solution to the problem of argument selection.

While this section is devoted to the theoretic foundations of probabilistic argumentation, we will provide a small empirical study to compare some of the proposed measures in Section 4.

### 3.1. Argument strength

As discussed above, we have two underlying measures which can serve as input for a measure of argument strength: the degree of support and the degree of possibility (recall Definition 4): str(*a*) = π(dsp(*a*), dps(*a*)) where π : {(*n, m*) ∈ [0, 1]^2^ ∣*n* ≤ *m*} → [0, 1]. As for π there are various straight-forward options. We list a few in Table 7. *Support* and *possibility* reflect the lower and upper probabilistic bounds represented by dsp and dps, while *mean* represents their mean. *Boosted support* follows the idea underlying Observation 1 according to which an argument *c* with dsp(*c*) < dps(*c*) should get a “boost” as compared to an argument *d* for which dsp(*d*) = dps(*d*) = dsp(*c*). The factor *m* ≥ 1 determines the magnitude of the boost, the lower *m* the more the lower bound dsp is boosted (where for *m* = 1 the boosted support is identical to dps). *Convex combination* follows a similar idea by letting the strength of an argument *a* be the result of a convex combination of dsp(*a*) and dps(*a*), where the parameter α determines how cautious an agent is: the higher α the less epistemic risk an agent is willing to take (where for α = 1 the convex combination is identical to dsp(*a*)). *Precision mean* is a qualification of mean in that it also considers the precision of an argument as a marker of strength (see Pfeifer, 2013). The precision of an argument *a* is given by 1−(dps(*a*)−dps(*a*)): the closer dsp(*a*) and dps(*a*) the more precise is *a*. The *precision mean* of an argument is the result of multiplying its mean with its precision. We note that this measure is in tension with the intuition behind Observation 1 in that it would measure the strength of hybrid higher than that of hybrid′, unlike *boosted support* or *convex combination*.

**Table 7:** Various notions of argument strength expressed as function of the degree of support and the degree of possibility of an argument.

**Name**	**π :(*x, y*) ↦ …**	**str(*a*) = π(dsp(*a*), dps(*a*)) = …**
Support	*x*	dsp(*a*)
Possibility	*y*	dps(*a*)
Mean	$\frac{x+y}{2}$	$mean\left(a\right)=\frac{\text{dsp}\left(a\right)+\text{dps}\left(a\right)}{2}$
Boosted support	$x+\frac{y-x}{m}$ (*m* ≥ 1)	${bst}_{m}\left(a\right)=\text{dsp}\left(a\right)+\frac{imprec\left(a\right)}{m}$
Convex combination	α·*x*+(1−α)·*y* (α ∈ [0, 1])	convex_α_(*a*) = α·dsp(*a*) + (1−α)·dps(*a*)
Precision mean	$\frac{x+y}{2}\cdot \left(1-\left(y-x\right)\right)$	precMean(*a*) = mean(*a*)·prec(*a*)

Clearly, some of the measures coincide for specific parameters (the proof can be found in Appendix A):

**Fact 3**. 1. mean(*a*) = bst_2_(*a*) = convex_.5_(*a*)
2. dsp(*a*) = convex_1_(*a*) and dps(*a*) = convex_0_(*a*) = bst_1_(*a*)
3. bst_*m*_(*a*) = convex_1−^1^/_*m*__(*a*) and convex_α_(*a*) = bst_^1^/_(1−α)__(*a*) (where α < 1).

*Proof*: Items 1 and 2 are trivial. We show Item 3. We have, on the one hand, ${bst}_{m}\left(a\right)=\text{dsp}\left(a\right)+\frac{\text{dps}\left(a\right)-\text{dsp}\left(a\right)}{m}=\text{dsp}\left(a\right)-\frac{\text{dsp}\left(a\right)}{m}+\frac{\text{dps}\left(a\right)}{m}=\left(1-{}^{1}{/}_{m}\right)\cdot \text{dsp}\left(a\right)+\left(1-\left(1-{}^{1}{/}_{m}\right)\right)\cdot \text{dps}\left(a\right)={convex}_{1-{}^{1}{/}_{m}}\left(a\right)$. On the other hand, convex_α_(*a*) = α·dsp(*a*) + (1 − α)dps(*a*) = dsp(*a*)+dps(*a*) − dps(*a*)·α − dsp(*a*)+dsp(*a*)·α = dsp(*a*) + (dps(*a*) − dsp(*a*))·(1 − α) = bst_^1^/_(1 − α)__(*a*).     □

**Example 8**. In Table 8, we apply the different argument strength measures to Examples 1 and 6.

**Table 8:** The strengths of arguments presented in Examples 5 and 6.

**Example**	**Argument**	**dsp(*a*)**	**dps(*a*)**	**mean(*a*)**	**bst_3_(*a*) resp.** **convex_⅔_(*a*)**	**precMean(*a*)**
Example 5	wave	0.7	0.7	0.7	0.7	0.7
	hybrid	0.7	1	0.85	0.8	0.595
	hybrid′	0.7	0.7	0.7	0.7	0.7
Example 6	*a*_1_	⅔	1	⅚	^7^/_9_	^5^/_9_
	*a*_2_	⅔	1	⅚	^7^/_9_	^5^/_9_
	*a*_3_	^8^/_9_	1	^17^/_18_	^25^/_27_	^68^/_81_
	*b*	⅔	⅔	⅔	⅔	⅔
	*c*	⅔	1	⅚	^7^/_9_	^5^/_9_

We now analyse the different strength measures in view of several properties, some of which may be considered desiderata.^6^ Table 9 offers an overview on which properties are satisfied for which measures.

- **Domain Restriction**. str(*a*) ∈ [dsp(*a*), dps(*a*)]. In the context of a given knowledge base, the degree of support represents a cautious estimation of the probability of the conclusion of *a* in view of its support, while the degree of possibility represents the most optimistic (in that it considers its possibility) estimation of its probability.
- **Precision**. If prec(*a*) = 1 then str(*a*) = dsp(*a*) = dps(*a*). This is a special case of Domain Restriction for cases in which the available information concerning *a* is precise.
- **Neutrality**. str(*a*) = 0.5 if prec(*a*) = 0. If prec(*a*) = 0, we have dsp(*a*) = 0 and dps(*a*) = 1. According to Neutrality we treat such cases as flipping an unbiased coin.
- **Moderation**. str(*a*) ≤ mean(*a*). Moderation is a cautious approach, putting more weight on the degree of support than the degree of possibility.

**Table 9:** Overview on the properties.

**Property**	**dsp(*a*)**	**dps(*a*)**	**mean(*a*)**	**bst_*m*_(*a*)**	**convex_α_(*a*)**	**precMean(*a*)**
Domain restriction^⊤^	✓	✓	✓	✓	✓	✗ [Example 10]
Precision^⊤^	✓	✓	✓	✓	✓	✓
Neutrality^⊤^	✗ [Example 9]	✗ [Example 9]	✓	✓ [*m* = 2]	✓ [α = 0.5]	✗
Moderation^⊤^	✓	✗ [Example 9]	✓	✓ [*m* ≥ 2]	✓ [α ≥ 0.5]	✓
Weak ep. sufficiency^♡^	✓	✓	✓	✓	✓	✗ [Example 10]
Strict ep. sufficiency	✗ [Example 9]	✗ [Example 9]	✓^⊤^	✗ [Example 9]°	✗ [Example 9]°	✗ [Example 10]
Ep. risk aversion	✓	✗ [Example 12]	✗ [Example 12]	✗ [Example 12]	✗ [Example 12]	✗ [Example 12]
Ep. risk tolerance	✗	✓ [Example 12]	✓ [Example 12]	✓ [Example 12]	✓ [α < 1]	✓
Upper compensation^•^	✗ [Example 9]	✓	✓^⊤^	✓	✓[α < 1]	✗ [Example 10]
Lower compensation^♠^	✓	✗ [Example 9]	✓	✓ [*m* ≥ 2]	✓ [α ≥ 0.5]	✗ [Example 10]
Precision sufficiency^†^	✓	✗ [Example 9]	✓	✓ [*m* ≥ 2]	✓ [α ≤ 0.5]	✓
Str. prec. sufficiency^†^	✓	✗ [Example 9]	✗ [Example 9]	✓ [*m* > 2]	✓ [α < 0.5]	✓
Precision necessity	✗ [Example 9]	✗ [Example 9]	✗ [Example 9]	✗ [Example 9]°	✗ [Example 9]°	✗ [Example 10]
Precision compensation	✓ [Proposition 4]	✗ [Example 9]	✓^⊤^	✗ [Example 9]°	✗ [Example 9]°	✓ [Proposition 4]
Counter^‡^	✓	✓	✓	✓	✓	✓
R-Weakening^⋆^	✓	✓	✓	✓	✓	✗ [Example 10]
L-Weakening^⋆^	✓	✓	✓	✓	✓	✓

(⊤) The proofs of these properties are trivial and therefore omitted. (♡) shown in Proposition 2. (♠) shown in Proposition 6. (†) shown in Proposition 3. (‡) shown in Proposition 7. (⋆) shown in Proposition 8. (•) shown in Proposition 5. (°) The counter-examples for dsp and *mean* apply in view of Fact 3. Proposition 2–8 and their proofs are presented in Appendix A (Supplementary material).

The following properties specify various ways the degrees of support and/or possibility are related to argument strength in terms of offering sufficient resp. necessary conditions. For the following properties let *a* ⊑ *b* iff dsp(*a*) ≤ dsp(*b*) and dps(*a*) ≤ dps(*b*). Let ⊏ be the strict version of ⊑, i.e., *a ⊏ b* iff *a* ⊑ *b* and *b* ⋢ *a*.

**Fact 4**. Let *a, b* be precise arguments (so, prec(*a*) = prec(*b*) = 1). If Precision holds for str, then: str(*a*) ≤ str(*b*) iff *a* ⊑ *b*.

- **Weak epistemic sufficiency**. str(*a*) ≤ str(*b*) if *a* ⊑ *b*.
- **Strict epistemic sufficiency**. str(*a*) < str(*b*) if *a ⊏ b*. Our Observation 1 follows the intuition of Strict epistemic sufficiency. In Example 5 we have hybrid ⊐ hybrid′ and therefore we expect also str(hybrid) > str(hybrid′).
- **Epistemic risk aversion**. dsp(*a*) ≤ dsp(*b*) if str(*a*) ≤ str(*b*). The criterion says that for *b* to be at least as strong as *a* it also has to have an at least as strong degree of support. The agent would take epistemic risk if it were to consider an argument *b* stronger than *a*, although *b* has less degree of support (but maybe more degree of possibility). The contrast case is expressed next.^7^
- **Epistemic risk tolerance**. It is possible that str(*a*) ≤ str(*b*) while dsp(*a*) > dsp(*b*).
- **Upper compensation**. str(*a*) > str(*b*) and mean(*a*) ≤ mean(*b*) implies dps(*a*) > dps(*b*). Choosing an argument *a* over *b* despite the fact that *b* has at least as high mean has to be compensated by *a* having a higher degree of possibility.
- **Lower compensation**. str(*a*) > str(*b*) and mean(*a*) ≤ mean(*b*) implies dsp(*a*) > dsp(*b*). Analogous to the previous criterion, except that the compensation is in terms of the degree of support.

The following criteria present various ways of considering precision a sign of argument quality. For instance, Pfeifer (2013) considers precision a central marker of strength.

- **Precision sufficiency**. If mean(*a*) = mean(*b*) and prec(*a*) ≥ prec(*b*) then str(*a*) ≥ str(*b*). If two arguments have the same mean, the one with more precision is better. The rationale is that the latter is supported by more informative evidence.
- **Strict precision sufficiency**. If mean(*a*) = mean(*b*) and prec(*a*) > prec(*b*) then str(*a*) > str(*b*).
- **Precision necessity**. str(*a*) ≥ str(*b*) implies prec(*a*) ≥ prec(*b*). An argument can only be at least as good as another one if its precision is at least as good.
- **Precision compensation**. str(*a*) > str(*b*) and mean(*a*) ≤ mean(*b*) implies prec(*a*) > prec(*b*). Choosing an argument *a* over *b* despite the fact that *b* has at least as high mean, has to be compensated by *a* having a higher precision.

Finally, we offer some criteria that relate arguments to other arguments in a logical way.

- **Counter**. If $\inf_{P\in \mathbb{P}}\left(P\left(∥Con\left(a\right){∥}_{C}\right)\right)=0$ and Con(*b*) = ¬Con(*a*), then str(*b*) ≥ str(*a*). If the conclusion of *a* has no probabilistic support in the knowledge base and *b* concludes the opposite, then *b* is at least as good as *a*.
- **R-Weakening**. If Sup(*a*) = Sup(*b*) and $Con\left(a\right){⊢}_{C}Con\left(b\right)$ then str(*b*) ≥ str(*a*). For two arguments with the same support the one with the logically weaker conclusion is at least as strong as the other argument. Clearly, its conclusion is more cautious.
- **L-Weakening**. If Sup(*a*) ⊇ Sup(*b*) and Con(*a*) = Con(*b*) then str(*a*) ≤ str(*b*). For two arguments with the same conclusions the argument which has more support is at most as strong as the other argument.

Before studying these properties for our different notions of argument strength, we observe some logical relations between some of them.

**Proposition 1**. For any argument strength measure str we have:

1. If str satisfies Domain restriction then it satisfies Precision.
2. If str satisfies Weak epistemic sufficiency, then it also satisfies R-weakening and L-weakening.

*Proof*: *Ad 1*. Trivial. *Ad 2*. Concerning R-weakening and L-weakening, observe that if *a* and *b* fulfill the requirements of the left hand side of R-weakening resp. of L-weakening, then *b* ⊒ *a*. So, by Weak epistemic sufficiency, str(*a*) ≤ str(*b*).     □

**Example 9** (Violation of properties for dsp, dps and mean.). An argument *a* with prec(*a*) = 0 is such that dsp(*a*) = 0 and dps(*a*) = 1. Clearly, *neutrality* is violated for dsp and dps. Such an argument also violates *moderation* for dps.

To illustrate other violations we give an example similar to Example 6. Let $𝕂=\langle \langle V_{p}:\left\{p_{1},p_{2}\right\},V_{l}:\left\{q_{1},q_{2},q_{3},q_{4}\right\}\rangle ,A:\text{sent}\left(V_{p}\right),C:\left\{\left(p_{1}\wedge p_{2}↔q_{1}\right),\neg \left(p_{1}\vee p_{2}\right)\to q_{2},p_{1}\to q_{4}\right\},\mathbb{P}:\left\{P\right\}\rangle$ with the probabilities as in Table 10. We note that mean(*a*_1_) > mean(*a*_2_) [resp. dsp(*a*_1_) > dsp(*a*_2_)] while dps(*a*_2_) > dps(*a*_1_) illustrating a violation of *lower compensation* for dps. Since prec(*a*_2_) = 0.1 < 1 = prec(*a*_1_) this also gives a counter-example for *precision compensation* and *necessity*, for dps. For a counter-example for *upper compensation* and dsp consider arguments *a*_3_ and *a*_5_: dsp(*a*_3_) < dsp(*a*_5_) and mean(*a*_5_) ≤ mean(*a*_3_), while dps(*a*_5_) < dps(*a*_3_). A counter-example for *strict epistemic sufficiency* and dps is given in view of dps(*a*_2_)≯dps(*a*_3_), although *a*_3_ ⊏ *a*_2_.

**Table 10:** Arguments and state space for the knowledge base $𝕂=\langle \langle V_{p}:\left\{p_{1},p_{2}\right\},V_{l}:\left\{q_{1},q_{2},q_{3},q_{4}\right\}\rangle ,A:\text{sent}\left(V_{p}\right)$, $C:\{\left(p_{1}\wedge p_{2}↔q_{1}\right),\neg \left(p_{1}\vee p_{2}\right)\to q_{2}$, *p*_1_ → *q*_4_}, ℙ : {*P*}〉, Example 9.

**State**	***p*_1_**	***p*_2_**	***P***	***q*_1_**	***q*_2_**	***q*_3_**	***q*_4_**	**Argument**	**dsp**	**dps**	**mean**	**precMean**
*s*_1_	0	0	0.1	0	1	◇	◇	*a*_1_ :〈{*p*_1_ ∧ *p*_2_}, *q*_1_〉	0.7	0.7	0.7	0.7
*s*_2_	0	1	0.1	0	◇	◇	◇	*a*_2_ :〈{¬(*p*_1_∨*p*_2_)}, *q*_2_〉	0.1	1	0.55	0.055
*s*_3_	1	0	0.1	0	◇	◇	1	*a*_3_ :〈∅, *q*_3_〉	0	1	0.5	0
*s*_4_	1	1	0.7	1	◇	◇	1	*a*_4_ :〈{*p*_1_}, *q*_4_〉	0.8	1	0.9	0.72
								*a*_5_ :〈{¬(*p*_1_ ∧ *p*_2_)}, ¬*q*_1_〉	0.3	0.3	0.3	0.3

*Strict epistemic sufficiency* and *precision necessity* for dsp is violated in view of hybrid and hybrid′ in Example 5, where dsp(hybrid) = dsp(hybrid′) while hybrid ⊐ hybrid′ and prec(hybrid) < prec(hybrid′).

Consider 𝕂=〈A:{p},C:∅,ℙ:{P}〉 where *P*(*p*) = 0.5 and the arguments *a* : 〈{*p*}, *p*〉 and *b* : 〈∅, ⊤〉. Then dsp(*a*) = 0.5 = dps(*a*) = mean(*a*) and prec(*a*) = 1, while dsp(*b*) = 0, dps(*b*) = 1, mean(*b*) = 0.5 and prec(*b*) = 0. The example represents a counter-example for (i) *precision necessity* for str ∈ {dps, mean}, (ii) *strict precision sufficiency* for str ∈ {dps, mean} and (iii) *precision sufficiency* for dps.

**Example 10** (Violation of properties for precision mean.). In Table 8, we have dsp(hybrid) = 0.7, dps(hybrid) = 1, while precMean(hybrid) = 0.595 (see Table 8). This shows that precMean does not satisfy *domain restriction*. Note that wave ⊑ hybrid and precMean(wave) = 0.7. So, we also have a counter-example for weak and strict *epistemic sufficiency*, as well as for *R-weakening*.

We consider the knowledge base $𝕂=\langle \langle V_{p}:\left\{p_{1},p_{2}\right\},V_{l}:\left\{q_{1},q_{2},q_{3},q_{4}\right\}\rangle ,A:\text{sent}\left(V_{p}\right),C:\left\{\neg \left(p_{1}\vee p_{2}\right)\to q_{1},\left(\neg p_{2}\vee p_{1}\right)\to q_{2},\neg p_{2}\to q_{3},\left(p_{1}\wedge p_{2}\right)\to \neg \left(q_{3}\vee q_{4}\right),\neg p_{1}\wedge p_{2}\to q_{4},\neg \left(p_{1}\vee p_{2}\right)\to \neg q_{4}\right\},\mathbb{P}:\left\{P\right\}\rangle$ with the probabilities and arguments in Table 11. For a counter-example for *upper* (resp. *lower*) *compensation* consider *a*_1_ and *a*_2_ (resp. *a*_3_). The arguments *a*_3_ and *a*_5_ also provide a counter-example for *precision necessity* since precMean(*a*_3_) > precMean(*a*_5_) while prec(*a*_5_) = 0.75 > prec(*a*_3_) = 0.6.

**Table 11:** Arguments and the state space for $𝕂=\langle \langle V_{p}:\left\{p_{1},p_{2}\right\},V_{l}:\left\{q_{1},q_{2},q_{3},q_{4}\right\}\rangle ,A:\text{sent}\left(V_{p}\right),C:\left\{\neg \left(p_{1}\vee p_{2}\right)\to q_{1},\left(\neg p_{2}\vee p_{1}\right)\to q_{2},\neg p_{2}\to q_{3},\left(p_{1}\wedge p_{2}\right)\to \neg \left(q_{3}\vee q_{4}\right),\neg p_{1}\wedge p_{2}\to q_{4},\neg \left(p_{1}\vee p_{2}\right)\to \neg q_{4}\right\},\mathbb{P}:\left\{P\right\}\rangle$ (see Example 10).

	***p*_1_**	***p*_2_**	***P***	***q*_1_**	***q*_2_**	***q*_3_**	***q*_4_**	**Argument**	**dsp**	**dps**	**mean**	**precMean**	**bst_1.5_**	**bst_2.5_**
*s*_1_	0	0	0.1	1	1	1	0	*a*_1_ :〈{*p*_1_}, *p*_1_〉	0.5	0.5	0.5	0.5	0.5	0.5
*s*_2_	0	1	0.4	◇	◇	◇	1	*a*_2_ :〈{¬(*p*_1_∨*p*_2_)}, *q*_1_〉	0.1	1	0.55	0.055	0.7	0.46
*s*_3_	1	0	0.25	◇	1	1	◇	*a*_3_ :〈{¬*p*_2_∨*p*_1_}, *q*_2_〉	0.6	1	0.8	0.48	0.867	0.76
*s*_4_	1	1	0.25	◇	1	0	0	*a*_4_ :〈{¬*p*_2_}, *q*_3_〉	0.35	0.75	0.55	0.33	0.62	0.51
								*a*_5_ :〈{¬*p*_1_ ∧ *p*_2_}, *q*_4_〉	0.4	0.65	0.525	0.394	0.567	0.5

**Example 11** (Violation of lower compensation, Boosted support, and Convex combination). In the knowledge base of Table 11 we have a counter-example for *lower compensation* and bst_*m*_ for *m* = 1.5. Note that bst_*m*_(*a*_2_) > bst_*m*_(*a*_4_) and mean(*a*_2_) ≤ mean(*a*_4_) while dsp(*a*_4_) > dsp(*a*_2_). In view of Fact 3 the example applies equally to convex_α_ for α = ⅓.

**Example 12** (Epistemic Risk Tolerance). We note that, in the example of Table 11, dps(*a*_2_) > dps(*a*_1_) [resp. mean(*a*_2_) > mean(*a*_1_)] (resp. bst_1.5_(*a*_2_) > bst_1.5_(*a*_1_)), while dsp(*a*_2_) < dsp(*a*_1_), demonstrating *epistemic risk tolerance* for dps [resp for mean] (resp. for bst_1.5_ and convex_⅔_). For precMean we consider arguments *a*_1_ and *a*_3_.

### 3.2. Naively applying argumentation semantics

Argumentation semantics aim at providing a rationale for selecting arguments for acceptance in discursive situations in which arguments and counter-arguments are exchanged. Some requirements are, for instance, that a selection does not contain conflicting arguments, or that a selection is such that any counter-argument to one of its arguments is attacked by some argument in the selection. In this section, we will gradually introduce new notions and observations based on a list of problems. Ultimately the critical discussion will lead to an improved account to be introduced in Section 3.3. In order to define argumentation semantics we first need a notion of argumentative defeat.

**Definition 5** (defeat types). Let 𝕂 be a knowledge base, str a strength measure, and *a, b* ∈ Arg(𝕂).

**rebuttal**: *a* rebuts *b* if (1) str(*a*) ≥ str(*b*) and (2) $Con\left(a\right){⊢}_{C}\neg Con\left(b\right)$.
**undercut**: *a* undercuts *b* if (1) str(*a*) ≥ str(*b*) and (2) $Con\left(a\right){⊢}_{C}\neg \wedge {Sup}^{\prime }$ for ∅ ≠ Sup′ ⊆ Sup(*b*).
**undercut**′: *a* undercuts′ *b* if (1) str(*a*) ≥ str(@(Sup(*b*))), where @(Sup(*b*)) = 〈Sup(*b*), ⋀ Sup(*b*)〉, and (2) $Con\left(a\right){⊢}_{C}\neg ⋀{Sup}^{\prime }$ for ∅ ≠ Sup′ ⊆ Sup(*b*).

**Lemma 1**. Suppose Weak Epistemic Sufficiency holds for str. Let *a, b* ∈ Arg(𝕂).

1. If $Con\left(a\right){⊢}_{C}Con\left(b\right)$ and Sup(*a*) = Sup(*b*) then str(*a*) ≤ str(*b*).
2. str(@(Sup(*a*))) ≤ str(*a*).
3. If Sup(*a*) ⊆ Sup(*b*) then str(@(Sup(*a*))) ≥ str(@(Sup(*b*))).
4. If *a* undercuts *b*, *a* also undercuts′ *b*.

*Proof*: *Ad 1*. Suppose $Con\left(a\right){⊢}_{C}Con\left(b\right)$ and Sup(*a*) = Sup(*b*). By Fact 2, dsp(*a*) = dsp(*b*) and dps(*b*) ≥ dps(*a*). By Weak Epistemic Sufficiency, str(*a*) ≤ str(*b*). *Ad 2*. This is a special case of item 1 since Sup(*a*) = Sup(@(Sup(*a*))) and $Con\left(@\left(Sup\left(a\right)\right)\right){⊢}_{C}Con\left(a\right)$. *Ad 3*. In this case $Con\left(@\left(Sup\left(b\right)\right)\right){⊢}_{C}Con\left(@\left(Sup\left(a\right)\right)\right)$. By Fact 2, dps(*a*) ≥ dps(*b*) and dsp(*a*) ≥ dsp(*b*). By Weak Epistemic Sufficiency, str(@(Sup(*a*))) ≥ str(@(Sup(*b*))). *Ad 4*. Suppose *a* undercuts *b*. In order to show that *a* undercuts′ *b* we only have to show that str(*a*) ≥ str(@(Sup(*b*))). Since str(*a*) ≥ str(*b*) this follows with Item 2.     □

We are now in a position to define argumentation frameworks and subsequently argumentation semantics.

**Definition 6** (AF). An *argumentation framework* based on a knowledge base 𝕂 is a pair 〈Arg(𝕂), def〉 where def is a (non-empty) set of defeat-types (as in Definition 5) for a given measure of argument strength str.

#### 3.2.1. Problem 1. Reinstatement and threshold selections

When selecting arguments for acceptance one may follow a naive “threshold-strategy” according to which one sets a threshold τ, say τ = 0.55, and simply selects all arguments which are stronger than τ (e.g., according to their degree of support, or another argument strength measure).^8^ This strategy, however, leads to various kinds of problems. One, illustrated in Example 16 below, is that following this strategy one may be left of with arguments whose conclusions form an inconsistent set. In this sense, the strategy selects too many arguments. On the other hand, this strategy does not validate a central principle from argumentation theory: reinstatement. It states that an argument which is defended by a set of accepted arguments, should also be accepted.

**Example 13** (Reinstatement). Consider the following knowledge base: $𝕂=\langle \langle V_{p}:\left\{w_{1},w_{2},w_{3}\right\},V_{l}:\left\{\text{scene},\text{home},\text{pub},\ldots \right\}\rangle ,A:\left\{w_{1},w_{2},w_{3}\right\},C:\left\{w_{1}\to \text{home},w_{2}\to \text{scene},w_{3}\to \text{pub},\neg \left(\text{scene }\wedge \text{ home}\right),\neg \left(\text{scene }\wedge \text{ pub}\right),\text{pub}\to \text{home}\right\},\mathbb{P}:\left\{P\right\}\rangle$. In our scenario we have 3 witnesses. Witness 1 states, among other things, that Mr. X was in his home town at the time of the murder (*w*_1_ → home), witness 2 states that Mr. X was at the scene of the murder (*w*_2_ → scene), and witness 3 that he was at the pub (*w*_3_ → pub). Mr. X cannot be both at the scene and in his home town. Also, the pub is located in Mr. X's home town. Witness 1 has a reliability of 0.82 (e.g., we estimate that she tells the truth in ^82^/_100_ cases), witness 2 of 0.91 and witness 3 of 0.6. After correcting the probabilities according to the states consistent with C (see Section 2.4) we obtain the ones listed in Table 12. There, we also state three key arguments *a*_1_, *a*_2_ and *a*_3_, their strength and the argumentation framework based on str = mean and rebuttal.

**Table 12:** State space, probabilities, and arguments for Example 13.

**State**	***w*_1_**	***w*_2_**	***w*_3_**	***P*(*s*_*i*_)**	** $P^{C}\left(s_{i}\right)$ **	**home**	**scene**	**pub**	**Argument**	**[dsp, dps]**	**mean**
*s*_1_	0	0	0	0.18 · 0.09 · 0.4	0.044	◇	◇	◇	*a*_1_	[0.506, 0.551]	0.528
*s*_2_	0	1	0	0.18 · 0.91 · 0.4	0.449	0	1	0	*a*_2_	[0.449, 0.494]	0.472
*s*_3_	1	0	0	0.82 · 0.09 · 0.4	0.202	1	0	◇	*a*_3_	[0.304, 0.551]	0.427
*s*_4_	1	0	1	0.82 · 0.09 · 0.6	0.304	1	0	1			
									AF	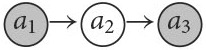	

We omit states that are incompatible with C. We calculate $P^{C}\left(s_{i}\right)$ by ^*P*(*s*_*i*_)^/_*e*_ where $e=P\left(∥⊤{∥}_{C}\right)=\sum_{i=1}^{4}P\left(s_{i}\right)=0.146$ (see Section 2.4). We have the arguments *a*_1_ :〈{*w*_1_}, pub〉, *a*_2_ :〈{*w*_2_}, scene〉 and *a*_3_ :〈{*w*_3_}, home〉. The argumentation framework on the right (bottom) is based on rebuttal and str = mean.

The strongest argument *a*_1_ is in favor of Mr. X being in his home town, which would clear Mr. X from suspicion. If we subscribe to this argument, however, the argument *a*_3_ for him being in the pub becomes quite reasonable, since its only attacker *a*_2_ (him being at the scene) is refuted. If we put ourselves in the investigative spirit of a detective working the case, it seems reasonable to select arguments *a*_1_ and *a*_3_ to form an investigative and/or explanatory hypothesis (despite the strength of *a*_3_ being below a threshold such as 0.5, both in terms of its degree of support or mean value). This hypothesis, may then lead us to the decision to investigate the question whether he was at the pub further in order to either substantiate or refute our stance.

**Observation 2** (Reinstatement). Argumentative reinstatement is not validated in naive threshold-based approaches for selecting arguments. This motivates other types of selections, since in specific reasoning scenarios, such as the formation of explanatory hypothesis, reinstatement is a reasonable argumentative principle.

Since threshold-based selection does not allow for reinstatement we will also study other selection types, in particular those introduced by Dung (1995) for abstract argumentation.

**Definition 7** (Argumentation Semantics, Dung, 1995). Given an 𝔸𝔽 = 〈Arg(𝕂), Def〉 and a set of arguments E⊆Arg(𝕂) we define

- E is *conflict-free* iff (E×E)∩Def=∅.
- E *defends* some *a* ∈ Arg(𝕂) iff for every defeater *b* of *a* there is a c∈E that defeats *b*.
- E is *admissible* iff E is conflict-free and it defends every a∈E.
- E is *complete* iff E is admissible and it contains every *a* ∈ Arg(𝕂) it defends.
- E is *grounded* iff it is the unique ⊆-minimal complete extension.
- E is α*-accepted* in case E={a∈Arg(𝕂)∣str(a)>α} (where α ∈ [0, 1], typically α = 0.5).
- E is *preferred* iff E is a ⊆-maximal complete extension.
- E is *stable* iff E is conflict-free and E∩Arg(𝕂) defeats every a∈Arg(𝕂)\E.

In the remainder of this section we show that naively applying these semantics to AFs leads to various problems. In the next section we present an alternative approach to resolve (some of) these issues.

Let us first highlight differences between the two types of defeat, rebut and undercut.

#### 3.2.2. Selecting arguments with inconsistent support with some semantics

**Example 14** (The possibility of inconsistent supports.). This example is similar to Example 3, where 𝕂=〈A:{p,¬p},C:{p→q},ℙ:{P}〉, except for the probability function *P* which is adjusted as described in Table 13 (left). On the right hand of the figure we describe the arguments and their respective strengths. It seems clear that the argument $a_{\bar{p}}$ in favor of ¬*p* is preferable to the argument *a*_*p*_ in favor of *p*, given that *P*(∥¬*p*∥) = ^4^/_7_ > ^3^/_7_ = *P*(∥*p*∥). What about *a*_*q*_ in favor of *q*, though? On the one hand, it is based on the assumption *p*, since only if *p* we know for certain that *q*. On the other hand, it comes with HOU, since for the case ¬*p* we are under-informed about *q*: *q* is possible (and so is ¬*q*). Thus, *q* seems to have more in its favor than ¬*q* and a reasoner committing to *q* seems not irrational, possibly even so when also selecting $a_{\bar{p}}$ and therefore committing to ¬*p*. Note that such a reasoner will not be committed to an inconsistent set of arguments (since {¬*p, q*} is consistent). In the following, we will show how some attack types support this kind of selections, while others do not.

**Observation 3** (Possibility of inconsistent supports.). In probabilistic argumentation, when situations of HOU arise, we can rationalize selections of arguments with mutually inconsistent supports (but consistent conclusions).

**Table 13:**
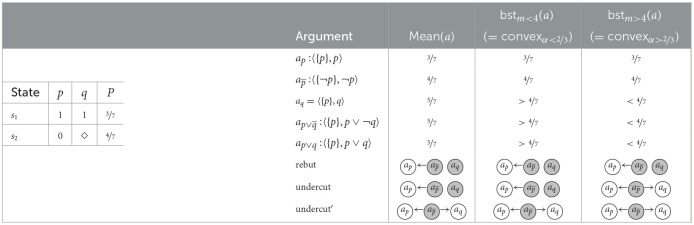
The state space, probabilities (left), arguments, and argumentation frameworks for different attack forms (right), Example 14 for 𝕂=〈A:{p,¬p},C:{p→q},ℙ:{P}〉.

It should be mentioned, though, that this observation is normatively cautious. We do not claim that from a normative philosophical perspective such selections are permissible (although they may be), we merely claim that agents are in a position to rationalize such selections. A formal framework modeling such selections is therefore at least attractive from a descriptive cognitive perspective (but possibly also from a normative philosophical perspective).

Let us now consider the different defeat-types in combination with our various argument strength measures, in order to see how they model the example. The underlying argumentation frameworks are illustrated in Table 13 (right, bottom).

**Rebut**. The argumentation framework based on rebut is in conformity with the rationale underlying Observation 3. Despite the fact that *a*_*q*_ is based on the support *p* and $a_{\bar{p}}$ attacks *a*_*p*_, $a_{\bar{p}}$, and *a*_*q*_ are selected.

**Undercut**. In case $str\left(a_{q}\right)>str\left(a_{\bar{p}}\right)$ (e.g., where str = mean or str = bst_*m*_ with *m* < 4, see Table 13), the latter is not sufficiently strong to defeat *a*_*q*_ leading to a selection analogous to the one based on rebut. Conceptually, however, undercut creates a tension in this and similar examples. While the rationale underlying undercut is that arguments with inconsistent supports should not both end up in the same selection, in our example they do since $a_{\bar{p}}$ is not strong enough to undercut *a*_*q*_ (while condition (2) of Definition 5 is met, condition (1) is not, which renders undercut unsuccessful in this case). This incoherence is resolved with our variant undercut′.

**Undercut**′. In contrast to undercut, for undercut′ for $a_{\bar{p}}$ to attack *a*_*q*_ it merely needs to be at least as strong as *a*_*p*_. Therefore, in all scenarios the attack is successfull (see right column in Table 13). Therefore, undercut′ does not allow for a selection of arguments with mutually inconsistent supports (We prove this impossibility in Section 3.4 after solving some other problems.).

#### 3.2.3. Problem 2: selecting arguments with inconsistent conclusions with rebut

When *only* working with rebut, we run into problems with inconsistent arguments, as the following example shows.

**Example 15** (Inconsistent conclusions with rebut.). Consider $𝕂=\langle \langle V_{p}:\left\{p\right\},V_{l}:\left\{q\right\}\rangle ,A:\left\{p,\neg p\right\},C:\emptyset ,\mathbb{P}:\left\{P\right\}\rangle$ where *P*(*p*) = 0.5. We have, for instance, the following arguments: *a*_⊤_ = 〈∅, ¬(*p* ∧ ¬*p*)〉, *a*_*p*_ = 〈{*p*}, *p*〉, $a_{\bar{p}}=\langle \left\{\neg p\right\},\neg p\rangle$, *a*_*q*_ = 〈{*p*, ¬*p*}, *q*〉 and $a_{\bar{q}}=\langle \left\{p,\neg p\right\},\neg q\rangle$. In an approach based on rebut, we get, for instance, a complete extension E containing the arguments *a*_⊤_, *a*_*p*_ and *a*_*q*_. The latter argument, or any argument for *q* based on 𝕂, is problematic in that it is based on an inconsistent support. Rebut does not effectively filter out such arguments. We also note that [dsp(*a*_⊤_), dps(*a*_⊤_)] = {1} while $\left[\text{dsp}\left(a_{q}\right),\text{dps}\left(a_{q}\right)\right]=\left[0,1\right]=\left[\text{dsp}\left(a_{\bar{q}}\right),\text{dps}\left(a_{\bar{q}}\right)\right]$. So, for any strength measure respecting Domain Restriction, $str\left(a_{⊤}\right)\geq str\left(a_{q}\right)=str\left(a_{\bar{q}}\right)$ and so *a*_⊤_ undercuts *a*_*q*_ and $a_{\bar{q}}$. This shows that with undercut-based attacks inconsistent arguments are “automatically” filtered out.

In order to deal with the problem of inconsistent arguments when using rebuts, we can either manually sort out inconsistent arguments (as proposed in Wu and Podlaszewski, 2014), or use inconsistency-undercuts (as proposed in Arieli and Straßer, 2020) in addition to rebuts.

**Inconsistency Undercut**. Where *a, b* ∈ Arg(𝕂), $Sup\left(b\right){⊢}_{C}⊥$, *a* = 〈∅, ¬ ⋀ Sup(*b*)〉 inconsistency-undercuts *b*.

**Lemma 2**. Let str satisfy Domain restriction. If *a* inconsistency undercuts *b*, then (i) *a* undercuts [resp. undercuts′] *b*, (ii) str(*a*) = 1, and (iii) there is no argument that defeats *a* (according to rebut, undercut, undercut′, or inconsistency undercut).

*Proof*: Suppose *a* inconsistency undercuts *b*. Since Sup(*a*) = ∅, by Domain restriction, $str\left(a\right)=\text{dsp}\left(a\right)=\text{dps}\left(a\right)=\inf_{P\in \mathbb{P}}P\left(∥⊤{∥}_{C}\right)=\sup_{P\in \mathbb{P}}P\left(∥⊤{∥}_{C}\right)=1$. This is (ii). For (i) it is sufficient to show that str(*a*) ≥ str(*b*). This follows trivially from (ii). For (iii) assume toward a contradiction that some *c* defeats *a*. Since Sup(*a*) = ∅, this cannot be an undercut, undercut′, or inconsistency undercut. Suppose *c* rebuts *a*. So, $Con\left(c\right){⊢}_{C}\wedge Sup\left(b\right)$ and therefore $Con\left(a\right){⊢}_{C}\neg Con\left(c\right)$. Moreover, $\emptyset {⊢}_{C}\neg Sup\left(c\right)$. So, dsp(*c*) = dps(*c*) = 0 since $∥Sup\left(c\right){∥}_{C}=∥◇Con\left(c\right){∥}_{C}=\emptyset$. Therefore, str(*c*) < str(*a*), a contradiction.     □

#### 3.2.4. Problem 3: (*n* > 2)-conflicts and selecting arguments with inconsistent conclusions

The following example illustrates that even in scenarios with exclusively precise probabilities (so, all arguments have precision 1) all discussed types of attack lead to problems.

**Example 16** ((*n* > 2)-conflicts and inconsistent selections.). Let $𝕂=\langle \langle V_{p}:\left\{p_{1},p_{2}\right\},V_{l}:\emptyset \rangle ,A:℘\left(\Gamma \right)\backslash \Gamma ,C:\emptyset ,\mathbb{P}:\left\{P\right\}\rangle$ where Γ = {*p*_1_, *p*_2_, ¬(*p*_1_ ∧ *p*_2_)}, *P* is given in Table 14 (right). There we also list arguments (left) with their corresponding strengths and an excerpt of the underlying argumentation framework (center), relative to any of the defeat-types, rebut, undercut and undercut′. As the reader can easily verify, there is a complete extension (highlighted) containing *a*_1_, *a*_2_, and *a*_*n*_. The problem with this selection is that it contains inconsistent conclusions, namely *p*_1_, *p*_2_, and ¬(*p*_1_ ∧ *p*_2_). The same problem occurs with α-selections for, e.g., α ≤ 0.54.

**Table 14:** The state space, probabilities (right), arguments and argumentation framework (left) for $𝕂=\langle \langle V_{p}:\left\{p_{1},p_{2}\right\},V_{l}:\emptyset \rangle ,A:℘\left(\Gamma \right)\backslash \Gamma ,C:\emptyset ,\mathbb{P}:\left\{P\right\}\rangle$, Γ = {*p*_1_, *p*_2_, ¬(*p*_1_ ∧ *p*_2_)}, and any of the defined strength measures str, Example 16.

**Argument**	str	**Attack diagram**				
*a*_1_ = 〈{*p*_1_}, *p*_1_〉	0.55	0.55	**State**	*p*_1_	*p*_2_	*P*
*a*_2_ = 〈{*p*_2_}, *p*_2_〉	0.55	0.55	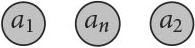 *s*_1_	0	0	0.05
*a*_*n*_ = 〈{¬(*p*_1_ ∧ *p*_2_)}, ¬(*p*_1_ ∧ *p*_2_)〉	0.85	0.85	↓ ↓ ↓ *s*_2_	0	1	0.40
*a*_*b*_ = 〈{*p*_1_, *p*_2_}, *p*_1_ ∧ *p*_2_〉	0.15	0.15	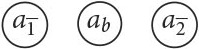 *s*_3_	1	0	0.40
$a_{\bar{1}}=\langle \left\{p_{2},\neg \left(p_{1}\wedge p_{2}\right)\right\},\neg p_{1}\rangle$	0.40	0.425	*s*_4_	1	1	0.15
$a_{\bar{2}}=\langle \left\{p_{1},\neg \left(p_{1}\wedge p_{2}\right)\right\},\neg p_{2}\rangle$	0.40	0.425				

In simple scenarios such as the one above, one may reasonably expect a reasoner to make a consistent selection of arguments.^9^

**Observation 4** (Inconsistency with regular AFs). Naively applying argumentation semantics in the context of probabilistic argumentation may lead to inconsistent selections, even for simple scenarios only including two probabilistic variables and no higher-order uncertainties. We consider this a serious problem, which we try to accommodate in the next section.

### 3.3. Using hyper-arguments: a refined method for argument selection

Given a knowledge based 𝕂, in order to enforce the consistency of the set of conclusion of a given complete extension we will make use of what we call *hyper-arguments* (collected in the set HArg(𝕂), see Definition 8 below), i.e., arguments written as [*a*_1_, …, *a*_*n*_] (where *a*_1_, …, *a*_*n*_ ∈ Arg(𝕂)). Hyper-arguments express the idea that if one were to accept each *a*_1_, …, *a*_*n*_ then one cannot accept a regular argument *b* ∈ Arg(𝕂) for which {*a*_1_, …, *a*_*n*_, *b*} is conflicting. For this a specific type of hyper-argument based defeat, so-called h-defeats, are introduced. From the argumentation theoretic perspective hyper-defeats express the meta-argumentative consideration that a reasoner should not commit to an inconsistent set of arguments. Therefore, hyper-arguments do not contribute to the content-level of a discussion, but rather they express constraints on argument selection.

In the following, we will make this idea formally precise, illustrate it with examples and study meta-theoretic properties in Section 3.4. As we will see, working both with normal and hyper-arguments, as well as both with h-defeats and defeats, suffices to ensure the consistency of the set of conclusions of complete extensions (and some other properties) and therefore avoids the problem pointed out in Observation 4.

**Definition 8** (Hyper-arguments.). Given a knowledge base 𝕂 and *a*_1_, …, *a*_*n*_ ∈ Arg(𝕂), [*a*_1_, …, *a*_*n*_] is a hyper-argument (based on 𝕂). We call *a*_1_, …, *a*_*n*_ the *components* of [*a*_1_, …, *a*_*n*_]. We let $Sup\left(\left[a_{1},\ldots ,a_{n}\right]\right)={⋃}_{i=1}^{n}Sup\left(a_{i}\right)$ and $Con\left(\left[a_{1},\ldots ,a_{n}\right]\right)={⋀}_{i=1}^{n}Con\left(a_{i}\right)$. We denote by HArg(𝕂) the set of all hyper-arguments *a* based on 𝕂.

In the following we will use the convention to use sub-scripted variables *a*_*i*_, *b*_*i*_, etc. for regular arguments (in Arg(𝕂)) and non-subscripted variables *a, b*, etc. for both regular arguments and hyper-arguments. We use ‘argument' as a generic term covering both regular and hyper-arguments.

Attacks are generalized to the level of hyper-arguments by letting, for instance, [*a*_1_, …, *a*_*n*_] *h-rebut* *b* in case $Con\left(\left[a_{1},\ldots ,a_{n}\right]\right){⊢}_{C}\neg Con\left(b\right)$. A hyper-argument is defeated resp. h-defeated by another regular argument resp. hyper-argument if one of its component arguments *a*_*i*_ is defeated resp. h-defeated (see Definition 9 below). While defeat is a relation on the domain Arg(𝕂) × (Arg(𝕂) ∪ HArg(𝕂)), h-defeat is a relation on the domain HArg(𝕂) × (HArg(𝕂) ∪ Arg(𝕂)).

**Definition 9** (h-defeat.). Let 𝕂 be a knowledge base. *h-defeats* define a relation on HArg(𝕂) × (Arg(𝕂) ∪ HArg(𝕂)). Let *a* = [*a*_1_, …, *a*_*n*_], *b* = [*b*_1_, …, *b*_*m*_] ∈ HArg(𝕂) and *c* ∈ Arg(𝕂).

- *a* *h-rebuts* *c* iff $Con\left(a\right){⊢}_{C}\neg Con\left(c\right)$.
- *a* *h-rebuts* *b* iff there is an *i* ∈ {1, …, *m*} for which *a* h-rebuts *b*_*i*_.
- *a* *h-undercuts* *c* iff $Con\left(a\right){⊢}_{C}\neg \wedge Sup\left(c\right)$.
- *a* *h-undercuts* *b* iff for some *i* ∈ {1, …, *m*}, *a* h-undercuts *b*_*i*_.

Note that unlike regular defeats, h-defeats do not consider argument strength. The reason is that h-defeats encode meta-argumentative considerations concerning the consistency of selections of arguments. For such considerations, argument strength is of no concern.

**Definition 10** (Regular defeats). Let 𝕂 be a knowledge base. *Defeats* define a relation on Arg(𝕂) × (Arg(𝕂) ∪ HArg(𝕂)) where the part on Arg(𝕂) × Arg(𝕂) is defined as in Definition 5, and some *a* ∈ Arg(𝕂) rebuts [resp. undercuts, undercuts′] some *b* = [*b*_1_, …, *b*_*n*_] ∈ HArg(𝕂) iff *a* rebuts [resp. undercuts, undercuts′] some component *b*_*i*_ of *b*.

**Fact 5**. Let 𝕂 be a knowledge base, *a* ∈ Arg(𝕂) ∪ HArg(𝕂) and *b* ∈ HArg(𝕂). *a* defeats [resp. h-defeats] *b* (according to rebut, undercut, undercut′ and consistency undercut) iff *a* defeats [resp. h-defeats] some component *b*_*i*_ of *b*.

Having defined regular and hyper-arguments and different notions of defeat among them, we are now in a position to generalize our notion of argumentation frameworks to include hyper-arguments.

**Definition 11** (Hyper AF, h-AF). A *hyper-argumentation framework* based on a knowledge base 𝕂 is a pair 〈〈Arg(𝕂), HArg(𝕂)), 〈Def, Hdef〉〉 where Def is a relation of regular defeat and Hdef a relation of hyper-defeat based on rebut and/or undercut and/or undercut′ and/or inconsistency undercut.

In the remainder, we consider three types of frameworks:

1. *rebut-based h-AFs*, where Def = {rebut, cons.undercut} and Hdef = {h-rebut}
2. *undercut-based h-AFs*, where Def = {undercut} and Hdef = {h-undercut}
3. *undercut*′*-based h-AFs*, where Def = {undercut′} and Hdef = {h-undercut}

Argumentation semantics are adjusted to the case with hyper-arguments as expected. We only need to adjust the notion of defense: defeats need to be counter-defeated, while h-defeats need to be counter-h-defeated.

**Definition 12** (Argumentation Semantics). Given an h-AF 𝔸𝔽 = 〈〈Arg(𝕂), HArg(𝕂)〉, 〈Def, Hdef〉〉 and a set of arguments E⊆Arg(𝕂)∪HArg(𝕂) we say

- E is *conflict-free* iff (E×E)∩(Def∪Hdef)=∅.
- E *defends* some *a* ∈ Arg(𝕂) ∪ HArg(𝕂) iff for every defeater [resp. h-defeater] *b* of *a* there is a c∈E that defeats [resp. h-defeats] *b*.
- E is *admissible* iff E is conflict-free and it defends every a∈E.
- E is *complete* iff E is admissible and it contains every *a* ∈ Arg(𝕂) ∪ HArg(𝕂) it defends.
- E is *preferred* iff E is a ⊆-maximal complete extension.
- E is *stable* iff E is conflict-free and E∩Arg(𝕂) defeats every a∈Arg(𝕂)\E.

Our definition requires that only h-defeats can defend from h-defeats. In Appendix C.1 (Supplementary material), we show that allowing regular defeats to defend from h-defeats leads to the same complete extensions (see Preposition 18).

**Figure 2:**
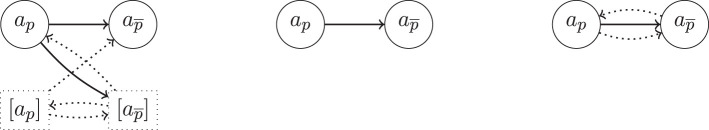
Illustration for Example 17. Dotted arrows indicate h-defeats, solid arrows regular defeats. **(Left)** Detailed presentation. **(Center)** Compact presentation omitting simple hyper-arguments. **(Right)** The presentation obtained by the variant defined in Appendix C.2 (Supplementary material).

**Example 17**. Let 𝕂=〈A:{p,¬p},C:∅,ℙ:{P}〉 where *P*(∥*p*∥) = 0.6 (and *P*(∥¬*p*∥) = 0.4). Let *a*_*p*_ = 〈{*p*}, *p*〉, $a_{\bar{p}}=\langle \left\{\neg p\right\},\neg p\rangle$. Let defeat be rebut (or undercut). In Figure 2 (left), we see an excerpt of an hyper-argumentation framework based on 𝕂. With the above definitions there is a slight redundancy in that every regular argument *a* has a hyper-argument [*a*] as counter-part. Note that *a*_*p*_ is defended from the hyper-attack by $\left[a_{\bar{p}}\right]$ by its hyper-argument counterpart [*a*_*p*_]. Unlike $a_{\bar{p}}$ and $\left[a_{\bar{p}}\right]$, *a*_*p*_ and [*a*_*p*_] are part of the unique preferred extension. Note that $a_{\bar{p}}$ and $\left[a_{\bar{p}}\right]$ cannot be defended from the defeat by *a*_*p*_.

In the following examples we will omit hyper-argumentative counterparts of regular arguments in the attack diagrams. For instance, Figure 2 (left) will be simplified to Figure 2 (center). In Appendix C.2 (Proposition 19), we show that it is possible to work without hyper-arguments of the form [*a*], i.e., to identify them with their regular counterparts. In our example this variant also results in Figure 2 (right).

We have omitted the grounded extension from Definition 12. Example 17 illustrates why. While we would expect *a*_*p*_ to be contained in the grounded extension, it is not since it is in need to be defended from the h-defeat by $\left[a_{\bar{p}}\right]$, but no non-attacked argument is able to do so. So, in many cases the grounded extension will not be informative since it will only contain arguments without h-attackers (e.g., those with tautological conclusions).^10^

**Figure 3:**
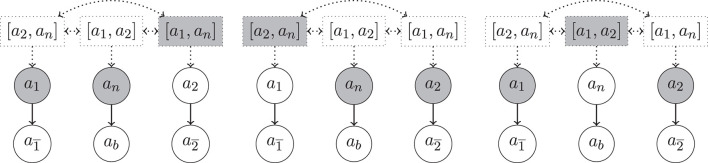
Excerpt of the hyper-argumentation frameworks for Example 18 with rebut, undercut, resp. undercut′. Highlighted are three preferred extensions (from **left** to **right**). As mentioned above, for the sake of compactness of presentation we identified simple hyper-arguments (i.e., hyper-arguments with only one component) with their component and omitted symmetric h-defeats between arguments whenever there are regular defeats present between their components (e.g., the h-defeat between [*a*_1_] and $\left[a_{\bar{1}}\right]$ is omitted since $a_{\bar{1}}$ is defeated by *a*_1_).

**Example 18** (Example 16 cont.). In Figure 3, we show excerpts of the argumentation frameworks for Example 16, now enriched with hyper-arguments. We have three preferred extensions, $E_{1}=\left\{a_{1},a_{n},\left[a_{1},a_{n}\right],\ldots \right\}$ (left), $E_{2}=\left\{a_{2},a_{n},\left[a_{2},a_{n}\right],\ldots \right\}$ (center) and $E_{3}=\left\{a_{1},a_{2},\left[a_{1},a_{2}\right],\ldots \right\}$ (right). We note that the problematic complete extension from Example 16, including arguments *a*_1_, *a*_2_ and *a*_*n*_ is not anymore admissible in the setup with hyper-arguments. One of the reasons is that the h-defeat from [*a*_2_, *a*_*n*_] on *a*_1_ cannot be defended. Indeed, the defeat from [*a*_2_, *a*_*n*_] expresses the consistency constraint that if we accept *a*_2_ and *a*_*n*_ then we shall not accept *a*_1_. We also note that neither of these three extensions is stable, e.g., $a_{\bar{2}}\notin E_{1}$ and $a_{\bar{2}}$ is also not defeated by $E_{1}$.

### 3.4. Rationality postulates for hyper-argumentation frameworks

We now study meta-theoretic properties of hyper-argumentation frameworks. Table 15 contains various properties, often called “rationality postulates” (see Caminada and Amgoud, 2007; Arieli et al., 2021). We will investigate these for our different attack types and for argument strength measures that satisfy Weak Epistemic Sufficiency and Domain Restriction. We consider two general scenarios: a *naive* one without hyperarguments (as discussed in Section 3.2) and a *hyper* one with hyperarguments. Table 16 provides an overview of our results. Proofs are provided in Appendix B (Supplementary material). We summarize:

**Observation 5** (Key observations.). Our results show that hyper-argument based probabilistic argumentation satisfies the desiderata discussed in Observations 1, 3, and 4.

Concerning Observation 1 we employ argument strength measures that satisfy weak epistemic sufficiency to do justice to the intuition that an argument such as hybrid is stronger than an argument such as wave due to the presence of higher-order uncertainty.

In order to model the intuition underlying Observation 3 one may use hyper-argumentation frameworks based on rebuttals: in such frameworks both $a_{\bar{p}}$ and *a*_*q*_ can be present in the same complete extension, without causing an inconsistent conclusion set.

Finally, we overcome the problem of the existence of complete extensions with inconsistent conclusion sets identified for regular argumentation frameworks in Observation 4: all of the studied hyper-argumentation frameworks satisfy the postulate of Indirect Consistency.

**Table 15:** A list of argumentation properties.

**Property**	**Definition**
Component closure	$\left[a_{1},\ldots ,a_{n}\right]\in E$ iff $a_{1},\ldots ,a_{n}\in E$.
Direct consistency	If $a_{1},a_{2}\in E$ then $Con\left(a_{1}\right),Con\left(a_{2}\right){⊬}_{C}⊥$.
Indirect consistency	If $a_{1},\ldots ,a_{n}\in E$ then $Con\left(a_{1}\right),\ldots ,Con\left(a_{n}\right){⊬}_{C}⊥$.
Weakening	If $a_{1}\in E$ and $Con\left(a_{1}\right){⊢}_{C}\phi$ then also $\langle Sup\left(a_{1}\right),\phi \rangle \in E$.
Support consistency	If $a_{1},\ldots ,a_{n}\in E$ then ${⋃}_{i=1}^{n}Sup\left(a_{i}\right){⊬}_{C}⊥$.
Dir. support closure	If $a_{1}\in E$ then for every *a*_2_ for which Sup(*a*_2_) ⊆ Sup(*a*_1_), $a_{2}\in E$.
Ind. support closure	If $a_{1},\ldots ,a_{n}\in E$ and *b*_1_ ∈ Arg(𝔸𝔽) is s. t. $Sup\left(b_{1}\right)\subseteq {⋃}_{i=1}^{n}Sup\left(a_{i}\right)$, then $b_{1}\in E$.
Logical closure	If $a_{1},\ldots ,a_{n}\in E$, $\langle {⋃}_{i=1}^{n}Sup\left(a_{i}\right),\phi \rangle \in E$ for all ϕ for which ${⋃}_{i=1}^{n}Sup\left(a_{i}\right){⊢}_{C}\phi$.

A property ℙ in the left column of the table holds for an argumentation semantics sem in case for every (hyper) argumentation framework 𝔸𝔽 and every sem-extension of 𝔸𝔽 the right column holds.

**Table 16:** Overview: rationality postulates.

**Method**	**Naive**	**Naive**	**Naive**	**Hyper**	**Hyper**	**Hyper**
**Arguments**	**Arg(𝕂)**	**Arg(𝕂)**	**Arg(𝕂)**	**HArg(𝕂)**	**HArg(𝕂)**	**HArg(𝕂)**
**Attack form(s)**	**Rebut**	**Undercut**	**Undercut^′^**	**Rebut**	**Undercut**	**Undercut^′^**
	**cons. u.cut**	**(cons. u.cut)**	**(cons. u.cut)**	**cons. u.cut**	**(cons. u.cut)**	**(cons. u.cut)**
Component closure	n.a.	n.a.	n.a.	✓ [Corollary 1]	✓ [Corollary 1]	✓ [Corollary 1]
Direct consistency	✓ [Proposition 11]	✓ [Proposition 11]	✓ [Proposition 11]	✓ [Proposition 11]	✓ [Proposition 11]	✓ [Proposition 11]
Indirect consistency	✗ [Example 16]	✗ [Example 16]	✗ [Example 16]	✓ [Proposition 12]	✓ [Proposition 13]	✓ [Proposition 13]
Weakening	✓[Proposition 10]	✓ [Proposition 9]	✓ [Proposition 9]	✓[Proposition 10]	✓ [Proposition 9]	✓ [Proposition 9]
Support consistency	✗ [Example 16]	✗ [Example 16]	✗ [Example 16]	✗^†^	✓ [Proposition 15]	✓ [Proposition 15]
Logical closure	✗ [Example 16]	✗ [Example 16]	✗ [Example 16]	✗^†^	✗^†^	✓^⋆^[Proposition 17]
Dir. support closure	✗ [Example 14]	✗ [Example 14]	✗ [Example 14]	✗^†^	✗^†^	✓[Proposition 14]
Ind. support closure	✗ [Example 16]	✗ [Example 16]	✗^†^	✗^†^	✗^†^	✓^⋆^[Proposition 16]

All propositions and corollaries are to be found in Appendix B (Supplementary material). Properties marked by ✓^⋆^ apply only to stable semantics. Counter-examples for other semantics can be found in Section 20, as well as counter-examples for properties marked with ^†^.

In the remainder of this section we illustrate the lack of some properties from Table 16 with examples. For this we first take another look at Example 14, this time with hyper-arguments.

**Figure 4:**
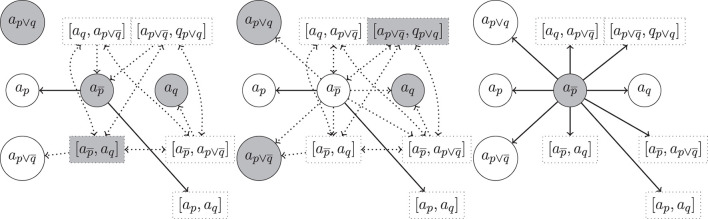
Hyper-argumentation frameworks for Example 14 with rebut **(left)**, undercut **(center)** and undercut′ **(right)** (where *m* < 4 in case of str = bst_*m*_ resp. $\alpha <\frac{2}{3}$ in case of str = convex_α_, see Table 13). Highlighted are preferred extensions. We use the same conventions as in Figure 3 to avoid clutter.

**Example 19** (Example 14 cont.). In Figure 4, we show excerpts of the argumentation frameworks for Example 14 for rebut (left), undercut (center), and undercut′ (right), now enriched with hyper-arguments. In each figure we highlight a preferred extension. We note that the one on the right is unique.

**Example 20** (Counter-examples, Rationality Postulates.). In Figures 3, 4, we observe the following violations of rationality postulates.

*Support Consistency*. Support consistency is violated for rebut in Figure 4 (left). There both $a_{\bar{p}}$ and *a*_*q*_ are contained in the given preferred extension, although $Sup\left(a_{\bar{p}}\right),Sup\left(a_{q}\right){⊢}_{C}⊥$.

*Logical Closure*. Logical closure is violated for rebut, undercut and undercut′ for complete and preferred extensions, as can be seen in Figure 3 (left). Although *a*_1_ and *a*_*n*_ are accepted in the given preferred extension, the argument $a_{\bar{2}}=\langle \left\{p_{1},\neg \left(p_{1}\wedge p_{2}\right)\right\},\neg p_{2}\rangle$ is not (it is and cannot be defended from the defeat by *a*_2_ since by Definition 12 a defense from a regular defeat must be in terms of a regular defeat and therefore the hyper-defeat of [*a*_1_, *a*_*n*_] on *a*_2_ is not sufficient to defend $a_{\bar{2}}$ from *a*_2_).

*Support Closure*. Direct support closure is violated for both rebuts and undercuts. For rebuts we have in the preferred extension of Figure 4 (left), *a*_*q*_ selected, but not *a*_*p*_ although Sup(*a*_*p*_) = Sup(*a*_*q*_). Similarly for undercuts, in Figure 4 (center). The violation of indirect support closure is an immediate consequence.

As for undercut′ and *indirect support closure* we consider Figure 3 (right): although *a*_1_ and *a*_2_ are selected, *a*_*b*_ = 〈{*p*_1_, *p*_2_}, *p*_1_ ∧ *p*_2_〉 is not since it cannot be defended from the undercut′ from *a*_*n*_. Note that the h-undercut on *a*_*n*_ by the selected [*a*_1_, *a*_2_] is not sufficient to defend *a*_*b*_ from a regular undercut′: Definition 12 requires a defense from a regular defeat in terms of a regular defeat (i.e., an undercut′ in this case).

## 4. Empirical study

In this section, we discuss a small empirical study we conducted on evaluating argument strength in the context of higher-order uncertainty.^11^ Our main objective was to investigate the following research questions:

**RQ1**. Is argument evaluation more context-sensitive than our logical model predicts? To answer this question we consider two reasoning contexts: an abstract one where participants have to reason about the probability to draw balls from an urn, and one practical medical context. In both scenarios, the participants face arguments of the same underlying logical form in our representation (see Appendices D, E in Supplementary material for details) but with different informal interpretations. For such arguments our model calculates the same degrees of support and possibilities, and therefore it predicts the same argument strengths. Similarly, we want to know whether across different contexts arguments of the same logical form are evaluated equally by our participants.

**RQ2**. How do the different argument strength measures from Section 3.1 predict the participants' answers? In particular, which values of the parameters *m* for bst_*m*_ resp. α for convex_α_ are empirically adequate (possibly relative to fixed reasoning contexts, see RQ1)?

**RQ3**. Which rationality postulates from Section 3.4 are met resp. violated by the participants' answers? In particular, is the intuition behind our Observation 1 empirically adequate?

**Figure 5:**
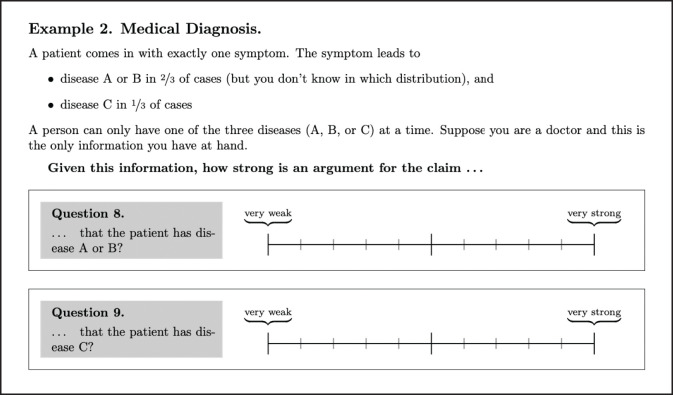
The second scenario of the questionnaire with two arguments.

The study was conducted in the context of three university seminars on the Bachelor and Master level of philosophy programs. Altogether 42 students participated. The questionnaire encompasses 19 questions and is structured into 3 reasoning scenarios. Each scenario comes with a number of arguments built on the basis of the available information. For each argument, the participants were asked to rate its strength in a scale with 10 subdivisions, reaching from *very weak* to *very strong* (see Figure 5 for two arguments in the context of the second scenario). We list all scenarios of the questionnaire in detail in Appendix C (Supplementary material).^12^

The three reasoning scenarios covered by the questionnaire are: (S1) one of the well-known Ellsberg scenarios (Ellsberg, 1961, see Example 2), (S2.1) a less abstract re-phrasing of the Ellsberg scenario in terms of a medical investigation, (S2.2) a variant of (S2.1) in which more emphasis is given to imprecise probabilistic information, as discussed in Section 2.3 (similar to Example 6).

Table 17 gives an overview on our results. We now evaluate our findings.

**Table 17:** Overview on the results from the empirical study on argument strength.

**Scenario**	**scenario S1 (Ellsberg)**					**Scenario 2.1 (Medical)**							**S2.2 (Impr. Prob.)**		
**Question**	**Q1**	**Q2**	**Q3**	**Q4**	**Q5**	**Q8**	**Q9**	**Q10**	**Q11**	**Q12**	**Q13**	**Q14**	**Q15**	**Q16**	**Q17**
Argument type	α	β	γ	δ	ϵ	δ	α	γ	β	γ	β	ϵ	α	λ	μ
dsp	⅓	0	⅓	⅔	1	⅔	⅓	⅓	0	⅓	0	1	⅓	⅓	0
dps	⅓	⅔	1	⅔	1	⅔	⅓	1	⅔	1	⅔	1	⅓	⅔	⅓
Claim type	at	at	∨	∨	∨	∨	at	∨	at	∨	at	∨	at	at	at
Average Strength	0.45	0.4	0.6	0.69	0.97	0.7	0.32	0.58	0.34	0.56	0.35	0.99	0.33	0.56	0.29
ordering	β < α < γ < δ < ϵ					α(<)β < γ < δ < ϵ							μ < α < λ		
Domain restr.	0.31	0.98	1	0.38	0.67	0.37	0.51	0.98	0.95	0.95	0.88	0.80	0.53	0.67	0.7
Moderation	0.36	0.36	0.64	0.45	-	0.41	0.76	0.78	0.68	0.78	0.61	-	0.76	0.5	0.29
Weak Ep. suff.	0.6					0.34							0.73		
Str. Ep. suff.	0.57					0.73							0.68		
Ep. risk tolerance	0.74					0.66							0.32		
Upper compensation	0.52		0.5			0.24	0.54	0.24	0.54	0.24	0.54		0.97		1
Lower compensation	0.79		0.88			0.88	0.63	0.88	0.63	0.88	0.63		0.97		1
Precision suff.	0.79		0.88			0.88	0.63	0.88	0.63	0.88	0.63		-		
Str. precision suff.	0.48		0.5			0.63	0.38	0.63	0.38	0.63	0.38		-		
Precision nec.	0.48		0.5			0.63	0.38	0.63	0.38	0.63	0.38		0.03		
Prec. compensation	0.48		0.5			0.63	0.38	0.63	0.38	0.63	0.38		0.03		1

Listed are the different scenarios (S1, S2.1, and S2.2) and their respective questions. Each question is concerned with the evaluation of the strength of an argument presented in an informal way. Some of these arguments share the same type w.r.t. their degrees of support and possibility (indicated by α, …, ϵ). Precise arguments (i.e, arguments *a* for which dsp(*a*) = dps(*a*)) are underlined. The exact logical form is presented in Appendix E (Supplementary material). We also list the type of claim (atomic “at” vs. disjunctive “∨”). Below we list the average strength assessment of the participants and an empirical evaluation of the properties from Section 3.1. In the first block we present properties concerned with arguments in isolation (Domain Restriction and Moderation, but note our cautious remarks concerning the evaluation of the survey with respect to these criteria in the main text). The second block concerns properties where arguments are compared. In these cases we analyse the two main scenarios S1 and S2 separately. For the desiderata weak/strong epistemic sufficiency and epistemic risk tolerance we compared the answers block-wise according to the scenarios 1, 2.1, and 2.2., i.e., 60% of participants validated weak epistemic sufficiency for all questions in scenario 1. For the rest of the criteria we picked out paradigmatic pairings of arguments. A number below two questions indicates that the arguments corresponding to those two questions were compared to each other according to the desideratum, e.g., the first value 0.52 for upper compensation means that w.r.t. the arguments in Q1 and Q2 52% of the participants answered in accordance with upper compensation. In scenario 2.1 whenever questions have the same number, it means those were compared to each other according to the desideratum, i.e., 24% of participants fulfilled upper compensation when comparing questions 8, 10, and 12 to each other. The hyphen ‘-' indicates that the criterion is not applicable.

### 4.1. Concerning RQ1

We first observe that the reasoning context is crucial for the assessment of argument strength. We note that scenarios 1 and 2.1 have the same formal structure and therefore our model predicts the same argument strength assessments for arguments of the same logical form (indicated by α, β, γ, δ and ϵ in Table 17). Indeed, within scenario 2.1, the evaluation of the strength of arguments of the same logical form (Q10 and Q12 resp. Q11 and Q13) remained relatively stable (max. variance is 0.02 between the mean values) among our participants. However, if we compare arguments of the same logical form between scenario 1 and scenario 2 we see clear differences. For instance for arguments of type α we have a difference of 0.13 in the mean, for arguments of type ϵ a difference of .02. In particular, the evaluation of α in the context of Q1 is 0.45 and in the context of Q9 it is 0.32. What is also striking is that for imprecise arguments there is basically no variance between the two scenarios. This asymmetry is surprising and we don't have an explanation for it.

### 4.2. Concerning RQ2

When averaging over all questions the optimal value for *m* is ≈ 2.05 and the one for α is ≈ 0.51. In view of this the mean measure is a good approximation of the empirical results. However, when zooming into the different types of arguments we observe that the *m* (resp. α) value is contextual, depending on where the [dsp, dps]-interval is situated. With Table 18 we observe the tendency that *m* grows the more the weight of the [dsp, dps]-interval moves toward 1. This means that the reasoning becomes more cautious resp. risk averse in such cases. For instance, the average strength estimation of arguments of type γ with [dsp, dps] = [⅓, 1] is 0.58 (closer to the dsp), while the average strength estimation of arguments of type μ with [dsp, dps] = [0, ⅓] is 0.29 (closer to the dps).

**Table 18:** Optimal *m* values for different argument types.

**Type**	**[dsp, dps]**	**Optimal *m***	**Average strength**
μ	[0, ⅓]	1.15	0.29
λ	[⅓, ⅔]	1.46	0.56
β	[0, ⅔]	1.84	0.36
γ	[⅓, 1]	2.63	0.58

### 4.3. Concerning RQ3

*Epistemic sufficiency* could be generally verified in the study.^13^ This reflects positively on our Observation 1 which can be considered empirically verified in view of our small study. Participants show typically *Risk tolerant* reasoning and therefore violated *Risk aversion*. *Upper compensation* could not convincingly be verified in our questionnaire. *Strict precision sufficiency, Precision necessity* and *compensation* only fare slightly better. In contrast, the acceptance rates for *Lower compensation* and for *Precision sufficiency* are in average high.

Before moving to *Domain restriction* and *Moderation*, we make two methodological remarks of caution. First, the scale of the questionnaire was not numerical and therefore it does not directly represent the interval [0, 1] in which our technical notions such as dsp, dps, etc. are measured. Therefore, a validation of criteria such as Domain restriction based on this questionnaire has to be interpreted with caution, since we naively mapped the interval in questionnaire to the interval [0, 1] (preserving scaling). Second, we interpreted the answers of the participants charitably, e.g., when evaluating Domain restriction we checked if the answer is “roughly” within the corresponding interval. Despite these methodological hurdles we consider the empirical study informative also for these criteria since it allows us to see discrepancies between the replies concerning logically equivalent arguments (indicated by types α, …, ϵ in Table 17) in different settings. We observe that *Domain Restriction* is violated for the types α and δ (even under a charitable interpretation of the answers). Interestingly for the imprecise arguments (so, arguments for which dsp(*a*) < dps(*a*)) *Domain Restriction* could be empirically verified. It is again the precise arguments as opposed to the imprecise ones, for which we find violations of *Moderation*. We see some divergence for arguments of type α between to two scenarios (in S2.1 and S2.2 Moderation is verified for α-type arguments, not in S1), while for arguments of type δ *Moderation* fails in more than 50% in both scenarios. One explanation may be that disjunctive claims may lead to over-estimating their associated probabilities. For imprecise arguments the observed shifting of the optimal *m*-value (see RQ2) has the effect that for arguments (such as γ) for which the weight of [dsp, dps]-interval is toward 1 a more risk-averse reasoning takes place and therefore *Moderation* is validated. The opposite applies to arguments (such as μ) for which the weight of [dsp, dps]-interval is toward 0, where *Moderation* is typically violated.

## 5. Discussion, conclusion, and outlook

Having presented our framework and results, we are in a position to situate this work in the context of probabilistic and formal argumentation. Our framework builds on Haenni's account of probabilistic argumentation (Haenni, 2009) and enhances it in several ways.

First, we adjust the representational form of argument to premise-conclusion pairs. This renders our approach a generalization of deductive argumentation (where the base logic is classical logic) and situates it in the tradition of formal argumentation. Also, our formalism generalizes Hunter's probabilistic argumentation (Hunter, 2013) in that it allows for HOU. Indeed, our framework inhabits the continuum between deductive argumentation (where $V_{p}=\emptyset$) and Hunter's probabilistic argumentation (where $V_{l}=\emptyset$). This mirrors a similar observation of Haenni for his original framework which is situated in the continuum between classical and probabilistic logic (Haenni, 2009, p. 165). Moreover, modeling arguments as premise-conclusion pairs (unlike Haenni, 2009) allows for capturing scenarios with different arguments that have the same conclusion but different supports and therefore possibly different strengths.

Second, we introduce several notions of argument strength and study them based on postulates. Our postulates are targeted at studying the role of HOU. This distinguishes it from the postulate-based study (Hunter, 2022) (which is based on probabilistic argumentation combined with defeasible logic). Argument strength has also been studied in Bayesian probabilistic argumentation (Hahn, 2020) and applied as a model of argumentative fallacies (Hahn and Oaksford, 2007). The idea that probability intervals can be utilized to model the actual reasoning of humans when confronted with scenarios such as Ellsberg's (see Example 2) is not new. Pfeifer and Pankka (2017) run an empirical study similar to ours to test the argument strength measure that has been dubbed *precision mean* in our paper (see also Pfeifer, 2013 for a motivation of this measure). Since the latter violates the desideratum *strict epistemic sufficiency* motivated in Observation 1 and instead opts for *precision sufficiency*, we included in our empirical study a scenario (S2.2) to test these postulates. Indeed, for the evaluation of argument strength our study indicates that *strict epistemic sufficiency* is more adequate than *precision sufficiency*. It seems to us that the latter more readily fits measures of argument *quality* than measures of *strength* (and, consequently, these two notions should be treated differently). Among the argument strength measures proposed in this paper are *convex combinations* of the degree of support and the degree of possibility. Convex combinations have also been used in formal epistemology as models of belief update, e.g., in context in which agents get information from other agents and an independent “truth signal” (Douven, 2010) or in which they are confronted with higher-order evidence (Henderson, 2021).

Third, we show how abstract argumentation semantics (Dung, 1995) can be applied to our framework given different (standard) notions of attack (versions of undercut and rebut). It is well-known from deductive argumentation that violations of rationality postulates can occur if one proceeds too naively. We proposed a solution based on *hyper-arguments*, which express consistency constraints. Given that our framework generalizes deductive argumentation and Hunter's probabilistic argumentation, the solution applies also there. In the context of probabilistic argumentation Dung's semantics are rarely applied. For example, Haenni (2009) does not propose any rationale for selecting arguments for selection, while Hunter (2013) uses threshold semantics. We consider Dung's semantics attractive for several reasons. First, they are widely applied and well-researched in formal argumentation (Baroni et al., 2018); second, being based on notions such as conflict-freeness and defendability, they are very intuitive; and third, they allow for reinstatement, a principle that is not (in general) validated by threshold semantics. The latter is in particular interesting when generating explanatory hypotheses (see Example 13 and Observation 2). In this context we note that it is sometimes distinguished between an epistemic and a constellations approach (Hunter, 2012). While in the former probabilities express a doxastic attitude toward arguments, in the latter they express how likely it is that arguments belong to and/or are relevant to a certain discursive situation. Our approach clearly belongs in the epistemic camp. We note that the interpretation of argument strength and defeat in structured non-probabilistic argumentation seems more in line with the epistemic approach and it is where reinstatement is often applied.^14^

The paper presents only a first step to systematically integrate reasoning with HOU in abstract argumentation. In future work, we intend to enhance the empirical study, both in terms of the number of participants and also in scope, by a stronger focus on the impact of context on argument strength, and by including questions of argument selection (e.g., is reinstatement used by participants when generating hypotheses and explanations?, etc.). Another application of our framework is to study in more detail reasoning in the context of multiple agents (e.g., considering testimony, higher-order evidence, and dialogue^15^). According to (Elkin and Wheeler, 2016; Elkin, 2021; Henderson, 2021) situations of peer disagreements and/or where higher-order evidence matters (e.g., evidence provided by expert panels, etc.) should not be modeled by naively aggregating beliefs, since this may overstate precision, but it should be modeled in terms of credal sets, i.e., in terms of HOU. Our framework provides some of the basic ingredients to model argumentation in such contexts. In the present work we restricted the focus on purely epistemic reasoning by not considering other practical utilities. A possible enhancement of our study is to widen the focus and incorporate decision theories under HOU (such as Gilboa and Schmeidler, 2004).

## Data availability statement

The raw data supporting the conclusions of this article will be made available by the authors, without undue reservation.

## Ethics statement

Ethical review and approval was not required for the study on human participants in accordance with the local legislation and institutional requirements. The patients/participants provided their written informed consent to participate in this study.

## Author contributions

All concepts and insights in the paper are a result of discussions and joint research between CS and LM. While it is hard to entangle individual contributions, CS's emphasis was on the theoretical foundation including meta-proofs, while LM's emphasis was on gathering and evaluating empirical data. All authors contributed to the article and approved the submitted version.

## Footnotes

^1^We note that following Keynes and Knight “uncertainty” is often used for non-probabilistic uncertainties in contradistinction to “risk” (which in this paper is first order uncertainty). For a discussion on the subtle differences in Knight and Keynes and for further discussion on the pairs of distinctions risk-vs-uncertainty and the related probabilistic-vs-non-probabilistic uncertainty (see O'Donnell, 2021). In contrast, here we use first order vs. higher-order uncertainty in place of risk vs. uncertainty.

^2^The focus point of the paper will be on strength measures that associate arguments with strength values in [0, 1], leading to linear strength orderings on the given set of arguments. In future work we will investigate broader notions allowing for incomparabilities between arguments.

^3^For work in non-probabilistic structured argumentation on hypothetical reasoning, see Beirlaen et al. (2018), Cramer and Dauphin (2019).

^4^Where the context disambiguates, we will omit the listing of the variables $V_{p}$ and $V_{l}$ to avoid clutter in the notation.

^5^Here we assume probabilistic independence of *p*_1_ and *p*_2_. If this assumption is given up we operate on the basis of ℙ = {*P*_λ, μ_∣λ ∈ [0, ⅔], μ ∈ [⅔ − λ, ⅓]} where *P*_λ,μ_ : *s*_1_ ↦ ⅓ − μ, *s*_2_ ↦ μ, *s*_3_ ↦ ⅔ − λ, *s*_4_ ↦ λ.

^6^The question of what properties are considered desired depends on the applications: if the application is to obtain a predictive mode of human reasoning these properties are in need of empirical verification (see Section 4).

^7^We note that Epistemic risk aversion is not very suitable for strength measures that linearly order arguments such as the ones studied in this paper. Indeed, dsp is the only of our measures that satisfies it.

^8^For instance, Hunter (2013) uses a threshold of 0.5 to define his *epistemic extensions*.

^9^However, there may be limitations to the requirement of consistency. As is well-known from cases such as the lottery paradox (Kyburg, 1961) or the preface paradox (Makinson, 1965), complex scenarios may give rise to inconsistent belief states, possibly even for rational reasoners. Although this is, as the reader may expect, a deep philosophical problem, see Douven and Williamson (2006) for a critical discussion. Clearly, though, in the simple examples included in our paper it should be considered irrational to hold inconsistent beliefs and we also don't expect it to be descriptionally adequate. A discussion of the mentioned paradoxical scenarios in the context of the formalism presented in this paper is left for future occasions.

^10^A similar observation can be made for stable semantics. While these need not exist in frameworks with odd defeat-cycles in regular AFs, the situation worsens in hyper-argumentation frameworks due to the presence of h-defeats (see Example 18). We include stable semantics nevertheless in Definition 12 since they satisfy some rationality postulates that don't hold for preferred semantics (see Table 16).

^11^We leave empirical studies concerning argument selection/semantics (Section 3) for a future occasion.

^12^In this study, we did not randomize the order of presentation for each participant in order to avoid priming.

^13^We note that the only case with a low acceptance rate is Scenario 2.1 for Weak epistemic sufficiency (34%). However, this value is due to the fact that there are 12 different pairwise argument combinations (e.g., Q8 and Q9, Q10 and Q11, etc.) in which participants could violate the criterion. The 34% feature only participants who validated weak epistemic sufficiency for every pair. If we consider each such pair separately, the acceptance rate per pair is rather high. In most of the pairs we see a very high acceptance rate for Weak epistemic sufficiency, only when comparing Q11 and Q13 resp. Q10 and Q12 our participants struggle. The reason is that for these particular pairs Weak epistemic sufficiency would demand equal strength attribution.

^14^Also in the context of the constellations approach imprecise probabilities have been utilized (see Fazzinga et al., 2022). It is particularly useful in cases in which our knowledge concerning the probabilities or arguments and attacks is limited.

^15^See Caminada (2018) for an overview on dialogical methods and abstract argumentation semantics.
