# Supplementary material for: Evaluating and selecting arguments in the context of higher order uncertainty
Source: Front Artif Intell. 2023 May 19;6:1133998. doi: 10.3389/frai.2023.1133998 (PMC10235603; doi:10.3389/frai.2023.1133998)
Supplement: Supplementary file 1 [file Data_Sheet_1.pdf]

Supplementary Material

In order for this appendix to be self-contained, we first recall the following definitions and results from the main paper.

Definition 9 (h-defeat.)**.** Let $\mathbb{K}$ be a knowledge base. *h-defeats* define a relation on $\mathsf{HArg}(\mathbb{K}) \times (\mathsf{Arg}(\mathbb{K}) \cup \mathsf{HArg}(\mathbb{K}))$. Let $a = [a_1, \ldots, a_n], b = [b_1, \ldots, b_m] \in \mathsf{HArg}(\mathbb{K})$ and $c \in \mathsf{Arg}(\mathbb{K})$.

- a *h-rebuts* c iff $\mathsf{Con}(a) \vdash_{\mathcal{C}} \neg\mathsf{Con}(c)$.
- a *h-rebuts* b iff there is an $i \in \{1, \ldots, m\}$ for which a h-rebuts b_i.
- a *h-undercuts* c iff $\mathsf{Con}(a) \vdash_{\mathcal{C}} \neg \bigwedge \mathsf{Sup}(c)$.
- a *h-undercuts* b iff for some $i \in \{1, \ldots, m\}$, a h-undercuts b_i.

Definition 12 (Argumentation Semantics)**.** Given an h-AF $\mathbb{AF} = \langle\langle \mathsf{Arg}(\mathbb{K}), \mathsf{HArg}(\mathbb{K})\rangle, \langle \mathsf{Def}, \mathsf{Hdef}\rangle\rangle$ and a set of arguments $\mathcal{E} \subseteq \mathsf{Arg}(\mathbb{K}) \cup \mathsf{HArg}(\mathbb{K})$ we say

- $\mathcal{E}$ is *conflict-free* iff $(\mathcal{E} \times \mathcal{E}) \cap (\mathsf{Def} \cup \mathsf{Hdef}) = \emptyset$.
- $\mathcal{E}$ *defends* some $a \in \mathsf{Arg}(\mathbb{K}) \cup \mathsf{HArg}(\mathbb{K})$ iff for every defeater [resp. h-defeater] b of a there is a $c \in \mathcal{E}$ that defeats [resp. h-defeats] b.
- $\mathcal{E}$ is *admissible* iff $\mathcal{E}$ is conflict-free and it defends every $a \in \mathcal{E}$.
- $\mathcal{E}$ is *complete* iff $\mathcal{E}$ is admissible and it contains every $a \in \mathsf{Arg}(\mathbb{K}) \cup \mathsf{HArg}(\mathbb{K})$ it defends.
- $\mathcal{E}$ is *preferred* iff $\mathcal{E}$ is a $\subseteq$-maximal complete extension.
- $\mathcal{E}$ is *stable* iff $\mathcal{E}$ is conflict-free and $\mathcal{E} \cap \mathsf{Arg}(\mathbb{K})$ defeats every $a \in \mathsf{Arg}(\mathbb{K}) \setminus \mathcal{E}$.

Fact 1. Let $a \in \mathsf{Arg}(\mathbb{K})$ and $\mathsf{Con}(a) \vdash_{\mathcal{C}} \phi$. Then 1(1) $\|\mathsf{Sup}(a)\| = \|\bigwedge \mathsf{Sup}(a)\| \subseteq \|\mathsf{Con}(a)\| \subseteq \|\Diamond\mathsf{Con}(a)\|$, (2) (1) $\|\mathsf{Sup}(a)\|_{\mathcal{C}} = \|\bigwedge \mathsf{Sup}(a)\|_{\mathcal{C}} \subseteq \|\mathsf{Con}(a)\|_{\mathcal{C}} \subseteq \|\phi\|_{\mathcal{C}}$, and (2) $\|\mathsf{Con}(a)\|_{\mathcal{C}} \subseteq \|\Diamond\mathsf{Con}(a)\|_{\mathcal{C}} \subseteq \|\Diamond\phi\|_{\mathcal{C}}$.

Fact 2. Let $a, b \in \mathsf{Arg}(\mathbb{K})$.

1. If $\mathsf{Sup}(a) \subseteq \mathsf{Sup}(b)$ then $\mathsf{dsp}(a) \geq \mathsf{dsp}(b)$.
2. If $\{\mathsf{Con}(a)\} \vdash_{\mathcal{C}} \mathsf{Con}(b)$ then $\mathsf{dps}(b) \geq \mathsf{dps}(a)$.

Fact 3.
1. $\mathsf{mean}(a) = \mathsf{bst}_2(a) = \mathsf{convex}_{.5}(a)$
2. $\mathsf{dsp}(a) = \mathsf{convex}_1(a)$ and $\mathsf{dps}(a) = \mathsf{convex}_0(a) = \mathsf{bst}_1(a)$
3. $\mathsf{bst}_m(a) = \mathsf{convex}_{1-1/m}(a)$ and $\mathsf{convex}_\alpha(a) = \mathsf{bst}_{1/(1-\alpha)}(a)$ (where $\alpha < 1$).

Fact 4. Let a, b be precise arguments (so, $\mathsf{prec}(a) = \mathsf{prec}(b) = 1$). If Precision holds for str, then: $\mathsf{str}(a) \leq \mathsf{str}(b)$ iff $a \sqsubseteq b$.

Fact 5. Let $\mathbb{K}$ be a knowledge base, $a \in \mathsf{Arg}(\mathbb{K}) \cup \mathsf{HArg}(\mathbb{K})$ and $b \in \mathsf{HArg}(\mathbb{K})$. a defeats [resp. h-defeats] b (according to rebut, undercut, undercut$'$ and consistency undercut) iff a defeats [resp. h-defeats] some component b_i of b

Proposition 1. For any argument strength measure str we have:

1. If str satisfies Domain restriction then it satisfies Precision.
2. If str satisfies Weak epistemic sufficiency, then it also satisfies R-weakening and L-weakening.

Lemma 1. Suppose Weak Epistemic Sufficiency holds for str. Let $a, b \in \mathsf{Arg}(\mathbb{K})$.

1. If $\mathsf{Con}(a) \vdash_{\mathcal{C}} \mathsf{Con}(b)$ and $\mathsf{Sup}(a) = \mathsf{Sup}(b)$ then $\mathsf{str}(a) \leq \mathsf{str}(b)$.
2. $\mathsf{str}(@(\mathsf{Sup}(a))) \leq \mathsf{str}(a)$.
3. If $\mathsf{Sup}(a) \subseteq \mathsf{Sup}(b)$ then $\mathsf{str}(@(\mathsf{Sup}(a))) \geq \mathsf{str}(@(\mathsf{Sup}(b)))$.
4. If a undercuts b, a also undercuts$'$ b.

Lemma 2. Let str satisfy Domain restriction. If a inconsistency undercuts b, then (i) a undercuts [resp. undercuts$'$] b, (ii) $\mathsf{str}(a) = 1$, and (iii) there is no argument that defeats a (according to rebut, undercut, undercut$'$, or inconsistency undercut).

A. TECHNICAL APPENDIX FOR SECTION 3.1

Proposition 2 (Weak Epistemic Sufficiency)**.** Weak epistemic sufficiency holds for $\mathsf{str} \in \{\mathsf{dsp}, \mathsf{dps}, \mathsf{mean}, \mathsf{bst}_m, \mathsf{convex}_\alpha \mid m \geq 1, \alpha \geq 0\}$.

PROOF. The proof is trivial for $\mathsf{str} \in \{\mathsf{dsp}, \mathsf{dps}, \mathsf{mean}\}$. We show it for bst_m (and in view of Fact 3 for convex_α). Suppose $a \sqsubseteq b$. Therefore, (†), $\mathsf{dsp}(a) \leq \mathsf{dsp}(b)$ and $\mathsf{dps}(a) \leq \mathsf{dps}(b)$. We have: $\mathsf{bst}_m(a) = \mathsf{dsp}(a) + \frac{\mathsf{dps}(a) - \mathsf{dsp}(a)}{m} = \mathsf{dsp}(a) \cdot \frac{m-1}{m} + \mathsf{dps}(a) \cdot \frac{1}{m} \leq^{[\text{by } \dagger]} \mathsf{dsp}(b) \cdot \frac{m-1}{m} + \mathsf{dps}(b) \cdot \frac{1}{m} = \mathsf{bst}_m(b)$. □

Proposition 3 (Precision sufficiency.)**.** Precision sufficiency holds for $\mathsf{str} \in \{\mathsf{dsp}, \mathsf{mean}, \mathsf{bst}_m, \mathsf{convex}_\alpha \mid m \geq 2, 0 \leq \alpha \leq .5\}$. Strict prec. sufficiency holds for $\mathsf{str} \in \{\mathsf{dsp}, \mathsf{bst}_m, \mathsf{convex}_\alpha \mid m > 2, 0 \leq \alpha < .5\}$.

PROOF. For dsp notice that $\mathsf{mean}(a) = \mathsf{mean}(b)$ and $\mathsf{prec}(a) \geq \mathsf{prec}(b)$ (resp. $\mathsf{prec}(a) > \mathsf{prec}(b)$) implies $\mathsf{dsp}(a) \geq \mathsf{dsp}(b)$ (resp. $\mathsf{dsp}(a) > \mathsf{dsp}(b)$). For mean and $\mathsf{precMean}$ precision sufficiency holds trivially. For $\mathsf{str} = \mathsf{bst}_m$ suppose (1) $\mathsf{mean}(a) = \mathsf{mean}(b)$ and (2) $\mathsf{prec}(a) \geq \mathsf{prec}(b)$. Then, $\mathsf{dsp}(a) = \mathsf{dsp}(b) + \epsilon$ and $\mathsf{dps}(a) = \mathsf{dps}(b) - \epsilon$ for some $\epsilon \geq 0$. Thus, $\mathsf{bst}_m(b) = (\mathsf{dsp}(a) - \epsilon) + \frac{\mathsf{dps}(a) + \epsilon - \mathsf{dsp}(a) + \epsilon}{m} = \mathsf{bst}_m(a) + \frac{2\epsilon}{m} - \epsilon = \mathsf{bst}_m(a) + \epsilon \cdot (\frac{2}{m} - 1)$. Since $m \geq 2$, $\mathsf{bst}_m(b) \leq \mathsf{bst}_m(a)$. The strict version and the case for $\mathsf{str} = \mathsf{convex}_\alpha$ are shown analogously. □

Proposition 4 (Precision Compensation)**.** Precision compensation holds for dsp and $\mathsf{precMean}$.

PROOF. Suppose that $\mathsf{mean}(a) \leq \mathsf{mean}(b)$. So, (1), $\mathsf{dsp}(a) + \mathsf{dps}(a) \leq \mathsf{dsp}(b) + \mathsf{dps}(b)$. Let $\mathsf{str} = \mathsf{dsp}$. Suppose that, (2), $\mathsf{dsp}(a) > \mathsf{dsp}(b)$. By (1) and (2), we have, (3), $\mathsf{dps}(a) < \mathsf{dps}(b)$. By (2) and (3), we have, $\mathsf{dps}(b) - \mathsf{dsp}(\mathsf{b}) > \mathsf{dps}(a) - \mathsf{dsp}(a)$. So, $\mathsf{prec}(a) = 1 - (\mathsf{dps}(a) - \mathsf{dps}(a)) > 1 - (\mathsf{dps}(b) - \mathsf{dsp}(b)) = \mathsf{prec}(b)$. The case for $\mathsf{str} = \mathsf{precMean}$ is analogous. □

Proposition 5 (Upper Compensation)**.** Upper compensation holds for dps, mean, bst_m and convex_α where $m \geq 1$ and $1 > \alpha$.

PROOF. Upper compensation holds trivially for dps. In view of Fact 3, we only show the case for $\mathsf{str} = \mathsf{bst}_m$ with $m < 1$ and $1 > \alpha > 0$. Suppose a and b are such that (1) $\mathsf{mean}(a) \leq \mathsf{mean}(b)$ and (2) $\mathsf{str}(a) > \mathsf{str}(b)$. Assume for a contradiction that (3) $\mathsf{dps}(a) \leq \mathsf{dps}(b)$. So, there is an $\epsilon \geq 0$ for which $\mathsf{dps}(a) = \mathsf{dps}(b) - \epsilon$. By (1) and (3),[1] $\mathsf{dsp}(a) - \epsilon \leq \mathsf{dsp}(b)$. By (2), $\mathsf{bst}_m(a) = \mathsf{dsp}(a) \cdot \frac{m-1}{m} + \frac{\mathsf{dps}(a)}{m} > \mathsf{bst}_m(b) = \mathsf{dsp}(b) \cdot \frac{m-1}{m} + \frac{\mathsf{dps}(b)}{m} = \mathsf{dsp}(b) \cdot \frac{m-1}{m} + \frac{\mathsf{dps}(a) + \epsilon}{m}$. So, $\mathsf{dsp}(a) \cdot \frac{m-1}{m} + \frac{\mathsf{dps}(a)}{m} > \mathsf{dsp}(b) \cdot \frac{m-1}{m} + \frac{\mathsf{dps}(a) + \epsilon}{m}$. Hence, $\mathsf{dsp}(a) - \frac{\epsilon}{m-1} > \mathsf{dsp}(b)$. In sum, $\mathsf{dsp}(b) \geq \mathsf{dsp}(a) - \epsilon > \mathsf{dsp}(a) - \frac{\epsilon}{m-1} > \mathsf{dsp}(b)$. This is a contradiction. □

[1] By (1), $\mathsf{dsp}(a) + \mathsf{dps}(a) \geq \mathsf{dsp}(b) + \mathsf{dps}(b)$ and by (3), $\mathsf{dsp}(a) + \mathsf{dps}(b) - \epsilon \geq \mathsf{dsp}(b) + \mathsf{dps}(b)$

Proposition 6 (Lower Compensation)**.** Lower compensation holds for dsp, mean, bst_m and convex_α, where $m \geq 2$ and $\alpha \geq 1/2$.

PROOF. The cases for dsp and mean are trivial. We show the case for bst_m and $m \geq 2$ (the case for convex_α and $\alpha \geq 1/2$ follows in view of Fact 3). Assume a, b represent a counter-instance of lower compensation. So, (1) $\mathsf{bst}_m(a) > \mathsf{bst}_m(b)$, (2) $\mathsf{mean}(a) \leq \mathsf{mean}(b)$, and (3) $\mathsf{dsp}(a) \leq \mathsf{dsp}(b)$. So, there is an $\epsilon \geq 0$ for which $\mathsf{dsp}(a) + \epsilon = \mathsf{dsp}(b)$. By (2), $2 \cdot \mathsf{mean}(a) = \mathsf{dsp}(a) + \mathsf{dps}(a) \leq \mathsf{dsp}(a) + \epsilon + \mathsf{dps}(b) = 2 \cdot \mathsf{mean}(b)$. So, (4), $\epsilon \geq \mathsf{dps}(a) - \mathsf{dps}(b)$. By (1), $\mathsf{dsp}(a) + \frac{\mathsf{dps}(a) - \mathsf{dsp}(a)}{m} > \mathsf{dsp}(a) + \epsilon + \frac{\mathsf{dps}(b) - \mathsf{dsp}(a) - \epsilon}{m}$. So, $\mathsf{dps}(a) > \epsilon \cdot m + \mathsf{dps}(b) - \epsilon$. So, $\mathsf{dps}(a) - \mathsf{dps}(b) > \epsilon(m-1)$. By (4), $\epsilon > 0$. So, $1 + \frac{\mathsf{dps}(a) - \mathsf{dps}(b)}{\epsilon} > m$. By (4), $2 > m$. □

Proposition 7. Counter holds for $\mathsf{str} \in \{\mathsf{dsp}, \mathsf{dps}, \mathsf{mean}, \mathsf{bst}_m, \mathsf{convex}_\alpha, \mathsf{precMean} \mid m \geq 1, \alpha \geq 0\}$.

PROOF. Suppose $\inf_{P \in \mathbb{P}}(P(\|\mathsf{Con}(a)\|)) = 0$ and $\neg\mathsf{Con}(a) = \mathsf{Con}(b)$. We first note three consequences of our suppositions. First, by Fact 1 we have $\mathsf{dsp}(a) = \inf_{P \in \mathbb{P}}(P(\|\mathsf{Sup}(a)\|)) \leq \inf_{P \in \mathbb{P}}(P(\|\mathsf{Con}(a)\|)) = 0$ and so, (1), $\mathsf{dsp}(a) = 0$. Second, since $\|\Diamond\mathsf{Con}(a)\| \subseteq \mathsf{states}(\mathcal{V}_p) \setminus \|\mathsf{Sup}(b)\|$, we have, (2), $\mathsf{dps}(a) = \sup_{P \in \mathbb{P}}(P(\|\Diamond\mathsf{Con}(a)\|)) \leq 1 - \inf_{P \in \mathbb{P}}(P(\|\mathsf{Sup}(b)\|)) = 1 - \mathsf{dsp}(b)$. Finally, (3), $\mathsf{dps}(b) = \sup_{P \in \mathbb{P}}(P(\|\Diamond\mathsf{Con}(b)\|)) = 1 - \inf_{P \in \mathbb{P}}(\|\mathsf{Con}(a)\|) = 1$.

We now show $\mathsf{str}(b) \geq \mathsf{str}(a)$. The case for $\mathsf{str} = \mathsf{dsp}$ [resp. $\mathsf{str} = \mathsf{dps}$] follows by (1) [resp. (3)]. Let $\mathsf{str} = \mathsf{mean}$. In view of (1) and (3) we have $\mathsf{mean}(a) = \frac{\mathsf{dsp}(a) + \mathsf{dps}(a)}{2} = \frac{0 + \mathsf{dps}(a)}{2} \leq \frac{\mathsf{dsp}(b) + \mathsf{dps}(b)}{2} = \frac{\mathsf{dsp}(b) + 1}{2} = \mathsf{mean}(b)$. Let now $\mathsf{str} = \mathsf{bst}_m$. In view of (1–3) we have $\mathsf{bst}_m(a) = \mathsf{dsp}(a) + \frac{\mathsf{dps}(a) - \mathsf{dsp}(a)}{m} = \frac{\mathsf{dps}(a)}{m} = \frac{1 - \mathsf{dsp}(b)}{m} \leq \mathsf{dsp}(b) + \frac{\mathsf{dps}(b) - \mathsf{dsp}(b)}{m} = \mathsf{dsp}(b) + \frac{1 - \mathsf{dsp}(b)}{m} = \mathsf{bst}_m(b)$. The case for $\mathsf{str} = \mathsf{convex}_\alpha$ is analogous. Let $\mathsf{str} = \mathsf{precMean}$. In view of (1), $\mathsf{precMean}(a) = \mathsf{dsp}(a) \cdot \mathsf{prec}(a) = 0 \leq \mathsf{precMean}(b)$. □

Proposition 8 (L/R-Weakening)**.** Right-Weakening holds for $\mathsf{str} \in \{\mathsf{dsp}, \mathsf{dps}, \mathsf{mean}, \mathsf{bst}_m, \mathsf{convex}_\alpha \mid m \geq 1, \alpha \geq 0\}$. Left-Weakening holds for $\mathsf{str} \in \{\mathsf{dsp}, \mathsf{dps}, \mathsf{mean}, \mathsf{bst}_m, \mathsf{convex}_\alpha, \mathsf{precMean} \mid m \geq 1, \alpha \geq 0\}$.

PROOF. We first consider R-weakening. Suppose, (1), $\mathsf{Sup}(a) = \mathsf{Sup}(b)$ and, (2), $\mathsf{Con}(a) \vdash_{\mathcal{C}} \mathsf{Con}(b)$. We have to show that $\mathsf{str}(b) \geq \mathsf{str}(a)$. This is Lemma 1 and Proposition 2.

We now consider L-weakening. Suppose $\mathsf{Sup}(a) \supseteq \mathsf{Sup}(b)$ and $\mathsf{Con}(a) = \mathsf{Con}(b)$. So, $\mathsf{dsp}(a) \leq \mathsf{dsp}(b)$ and $\mathsf{dps}(a) = \mathsf{dps}(b)$. We now show that $\mathsf{str}(a) \leq \mathsf{str}(b)$.

The cases $\mathsf{str} = \mathsf{dsp}$, $\mathsf{str} = \mathsf{dps}$ and $\mathsf{str} = \mathsf{mean}$ follow immediately. Consider $\mathsf{str} = \mathsf{bst}_m$. We have $\mathsf{bst}_m(a) = \mathsf{dsp}(a) + \frac{\mathsf{dps}(a) - \mathsf{dsp}(a)}{m} = \mathsf{dsp}(a) \cdot \frac{m-1}{m} + \frac{\mathsf{dps}(a)}{m} = \mathsf{dsp}(a) \cdot \frac{m-1}{m} + \frac{\mathsf{dps}(b)}{m} \leq \mathsf{dsp}(b) \cdot \frac{m-1}{m} + \frac{\mathsf{dps}(b)}{m} = \mathsf{bst}_m(b)$. The case for $\mathsf{str} = \mathsf{convex}_\alpha$ is analogous. Finally, let $\mathsf{str} = \mathsf{precMean}$. We first note that $\mathsf{prec}(a) = 1 - (\mathsf{dps}(a) - \mathsf{dsp}(a)) \leq 1 - (\mathsf{dps}(b) - \mathsf{dsp}(b)) = \mathsf{prec}(b)$. We have already shown that $\mathsf{mean}(a) \leq \mathsf{mean}(b)$ and therefore $\mathsf{precMean}(a) = \mathsf{mean}(a) \cdot \mathsf{prec}(a) \leq \mathsf{mean}(b) \cdot \mathsf{prec}(b) = \mathsf{precMean}(b)$. □

B. TECHNICAL APPENDIX FOR SECTION 3.4

The following results apply to all knowledge bases $\mathbb{K}$, all hyper-argumentation frameworks $\mathbb{AF} = \langle\langle \mathsf{Arg}(\mathbb{K}), \mathsf{HArg}(\mathbb{K})\rangle, \langle \mathsf{def}, \mathsf{Hdef}\rangle\rangle$ which are rebut-based (def consists of rebut and consistency undercut, and hdef is h-rebut), undercut-based (def is undercut and Hdef is h-undercut), or undercut′-based (def is undercut′ and Hdef is h-undercut). In the context of the following results, unless otherwise stated, $\mathcal{E}$ is a complete extension of $\mathbb{AF}$.

Lemma 3. (1) If $a_1, \ldots, a_n \in \mathcal{E}$ then $[a_1, \ldots, a_n] \in \mathcal{E}$. (2) If $[a_1, \ldots, a_n] \in \mathcal{E}$ then $[a_2, \ldots, a_n] \in \mathcal{E}$. (3) If $[a_1, \ldots, a_n] \in \mathcal{E}$ then $a_1, \ldots, a_n \in \mathcal{E}$.

PROOF. Ad 1. Let $a_1, \ldots, a_n \in \mathcal{E}$. Suppose some $b \in \mathsf{Arg}(\mathbb{K}) \cup \mathsf{HArg}(\mathbb{K})$ defeats [resp. h-defeats] $[a_1, \ldots, a_n]$. By Fact 5, there is an $i \in \{1, \ldots, n\}$ for which b defeats [resp. h-defeats] a_i. Since $\mathcal{E}$ is admissible, there is a $c \in \mathcal{E}$ that defeats [resp. h-defeats] b. This shows that $[a_1, \ldots, a_n]$ is defended by $\mathcal{E}$. By the completeness of $\mathcal{E}$, $[a_1, \ldots, a_n] \in \mathcal{E}$.

Ad 2. Let $[a_1, \ldots, a_n] \in \mathcal{E}$. Suppose $b \in \mathsf{Arg}(\mathbb{K}) \cup \mathsf{HArg}(\mathbb{K})$ defeats [resp. h-defeats] $[a_2, \ldots, a_n]$. By Fact 5, b defeats [resp. h-defeats] $[a_1, \ldots, a_n]$. By the admissibility of $\mathcal{E}$, b is defeated [resp. h-defeated] by $\mathcal{E}$. So $\mathcal{E}$ defends $[a_2, \ldots, a_n]$. By the completeness of $\mathcal{E}$, $[a_2, \ldots, a_n] \in \mathcal{E}$.

Ad 3. Let $[a_1, \ldots, a_n] \in \mathcal{E}$. We paradigmatically show that $a_1 \in \mathcal{E}$. Suppose some $b \in \mathsf{Arg}(\mathbb{K}) \cup \mathsf{HArg}(\mathbb{K})$ defeats [resp. h-defeats] a_1. By Fact 5, b defeats [resp. h-defeats] $[a_1, \ldots, a_n]$. Since $\mathcal{E}$ is admissible, there is a $c \in \mathcal{E}$ that defeats [resp. h-defeats] b. So, a_1 is defended by $\mathcal{E}$ and by the completeness of $\mathcal{E}$, $a_1 \in \mathcal{E}$. □

Corollary 1 (Component Closure)**.** $a_1, \ldots, a_n \in \mathcal{E}$ iff $[a_1, \ldots, a_n] \in \mathcal{E}$.

The proofs of the following three propositions apply both to regular and hyper-argument frameworks (for regular frameworks the reader may simply ignore h-arguments and h-defeats).

Proposition 9 (Weakening for Undercut and Undercut′)**.** If $a_1 \in \mathcal{E}$ and $\mathsf{Con}(a_1) \vdash_{\mathcal{C}} \phi$, then $a_1' = \langle \mathsf{Sup}(a_1), \phi \rangle \in \mathcal{E}$.

PROOF. Let $a_1 \in \mathcal{E}$, $\mathsf{Con}(a_1) \vdash_{\mathcal{C}} \phi$, and $a_1' = \langle \mathsf{Sup}(a_1), \phi \rangle$. We show the case for undercut, the case for undercut′ is similar and left to the reader. Suppose b undercuts [resp. h-undercuts] a_1'. So, $\mathsf{Con}(b) \vdash_{\mathcal{C}} \neg \bigwedge \mathsf{Sup}(a_1)$. By Lemma 1, $\mathsf{str}(a_1) \leq \mathsf{str}(a_1')$. So, b undercuts [resp. h-undercuts] a_1. By the admissibility of $\mathcal{E}$, b is undercut [resp. h-undercut] by $\mathcal{E}$. So, $\mathcal{E}$ defends a_1'. By the completeness of $\mathcal{E}$, $a_1' \in \mathcal{E}$. □

Proposition 10 (Weakening for Rebut)**.** If $a_1 \in \mathcal{E}$ and $\mathsf{Con}(a_1) \vdash_{\mathcal{C}} \phi$, then also $a_1' = \langle \mathsf{Sup}(a_1), \phi \rangle \in \mathcal{E}$.

PROOF. Suppose some b rebuts [resp. h-rebuts] a_1'. So, $\mathsf{Con}(b) \vdash_{\mathcal{C}} \neg\phi$ and therefore $\mathsf{Con}(b) \vdash_{\mathcal{C}} \neg\mathsf{Con}(a_1)$. By Lemma 1, $\mathsf{str}(a_1) \leq \mathsf{str}(a_1')$. So b rebuts [resp. h-rebuts] a_1. By the admissibility of $\mathcal{E}$, b is rebutted [resp. h-rebutted] by $\mathcal{E}$. Assume towards a contradiction that some b consistency-undercuts a_1'. Thus, b also consistency-undercuts a_1. But by Lemma 2, $\mathcal{E}$ cannot defend itself against b, which is a contradiction to the admissibility of $\mathcal{E}$.

So, altogether we have shown that $\mathcal{E}$ defends a_1' and by the completeness of $\mathcal{E}$, $a_1' \in \mathcal{E}$. □

Proposition 11 (Direct Consistency for Rebut, Undercut, and Undercut′)**.** If $a_1, a_2 \in \mathcal{E}$ then $\mathsf{Con}(a_1), \mathsf{Con}(a_2) \nvdash_{\mathcal{C}} \bot$.

PROOF. We present the proof for undercut and leave the similar proofs for rebut and undercut′ to the reader. Suppose $\mathsf{Con}(a_1), \mathsf{Con}(a_2) \vdash_{\mathcal{C}} \bot$, where $a_1, a_2 \in \mathcal{E}$. Without loss of generality suppose that $\mathsf{str}(a_1) \geq \mathsf{str}(a_2)$. Note that $\mathsf{Con}(a_1) \vdash_{\mathcal{C}} \neg \bigwedge \mathsf{Sup}(a_2)$ (since $\mathsf{Con}(a_1) \vdash_{\mathcal{C}} \neg\mathsf{Con}(a_2)$ and $\bigwedge \mathsf{Sup}(a_2) \vdash_{\mathcal{C}} \mathsf{Con}(a_2)$). Consider the argument $a_3 = \langle \mathsf{Sup}(a_1), \neg \bigwedge \mathsf{Sup}(a_2) \rangle$. By Proposition 9, $a_3 \in \mathcal{E}$. By Lemma 1, $\mathsf{str}(a_3) \geq \mathsf{str}(a_1)$ and therefore a_3 undercuts a_2. This contradicts the conflict-freeness of $\mathcal{E}$. □

We now move to properties which exclusively hold for the hyper-argument frameworks.

Proposition 12 (Indirect Consistency for Rebut)**.** If $a_1, \ldots, a_n \in \mathcal{E}$, then $\mathsf{Con}(a_1), \ldots, \mathsf{Con}(a_n) \nvdash_{\mathcal{C}} \bot$.

PROOF. Let $a_1, \ldots, a_n \in \mathcal{E}$. By Corollary 1, $[a_1, \ldots, a_n] \in \mathcal{E}$. Assume for a contradiction that $\mathsf{Con}(a_1), \ldots, \mathsf{Con}(a_n) \vdash_{\mathcal{C}} \bot$. Then $[a_1, \ldots, a_n]$ h-rebuts a_1. This contradicts the conflict-freeness of $\mathcal{E}$. So, $\mathsf{Con}(a_1), \ldots, \mathsf{Con}(a_n) \nvdash_{\mathcal{C}} \bot$. □

Proposition 13 (Indirect Consistency for Undercut and Undercut′)**.** If $a_1, \ldots, a_n \in \mathcal{E}$, then $\mathsf{Con}(a_1), \ldots, \mathsf{Con}(a_n) \nvdash_{\mathcal{C}} \bot$.

PROOF. Let $a_1, \ldots, a_n \in \mathcal{E}$. Assume for a contradiction that $\mathsf{Con}(a_1), \ldots, \mathsf{Con}(a_n) \vdash_{\mathcal{C}} \bot$. Thus, $\bigcup_{i=2}^{n} \mathsf{Sup}(a_i) \vdash_{\mathcal{C}} \neg \bigwedge \mathsf{Sup}(a_1)$. By Lemma 3, $[a_2, \ldots, a_n] \in \mathcal{E}$. Hence, $[a_2, \ldots, a_n]$ h-undercuts a_1. This contradicts the conflict-freeness of $\mathcal{E}$. □

We now move to properties which hold only for undercut′.

Proposition 14 (Direct Support Closure, Undercut′)**.** If $a_1 \in \mathcal{E}$ and $\mathsf{Sup}(a_2) \subseteq \mathsf{Sup}(a_1)$, then $a_2 \in \mathcal{E}$.

PROOF. Let $a_1 \in \mathcal{E}$ and $a_2 \in \mathsf{Arg}(\mathbb{AF})$ such that $\mathsf{Sup}(a_2) \subseteq \mathsf{Sup}(a_1)$. By Lemma 1, $\mathsf{str}(@(\mathsf{Sup}(a_1))) \leq \mathsf{str}(@(\mathsf{Sup}(a_2)))$. Suppose b undercuts′ [resp. h-undercuts] a_2. Thus, $\mathsf{Con}(b) \vdash_{\mathcal{C}} \neg \bigwedge \mathsf{Sup}(a_2)$, and in the case of undercut′, $\mathsf{str}(b) \geq \mathsf{str}(@(\mathsf{Sup}(a_2)))$. Also, $\mathsf{Con}(b) \vdash_{\mathcal{C}} \neg \bigwedge \mathsf{Sup}(a_1)$ and, in the case of undercut′, $\mathsf{str}(b) \geq \mathsf{str}(@(\mathsf{Sup}(a_1)))$. Thus, b undercuts′ [resp. h-undercuts] a_1. By the admissibility of $\mathcal{E}$, $\mathcal{E}$ undercuts′ [resp. h-undercuts] b. So, $\mathcal{E}$ defends a_2. By the completeness of $\mathcal{E}$, $a_2 \in \mathcal{E}$. □

Proposition 15 (Support Consistency for Undercut and Undercut′)**.** If $a_1, \ldots, a_n \in \mathcal{E}$, then $\bigcup_{i=1}^{n} \mathsf{Sup}(a_i) \nvdash_{\mathcal{C}} \bot$.

PROOF. Let $a_1, \ldots, a_n \in \mathcal{E}$. Assume for a contradiction that $\bigcup_{i=1}^{n} \mathsf{Sup}(a_i) \vdash_{\mathcal{C}} \bot$. Thus, $[@(\mathsf{Sup}(a_2)), \ldots, @(\mathsf{Sup}(a_n))]$ h-undercuts a_1. By the admissibility of $\mathcal{E}$, some $c \in \mathcal{E}$ h-undercuts $[@(\mathsf{Sup}(a_2)), \ldots, @(\mathsf{Sup}(a_n))]$. So, there is an $i \in \{2, \ldots, n\}$ for which c h-undercuts a_i. Since $\mathsf{Sup}(@(\mathsf{Sup}(a_i))) = \mathsf{Sup}(a_i)$, c also h-undercuts a_i. Since $a_i \in \mathcal{E}$ this is a contradiction to the conflict-freeness of $\mathcal{E}$. □

Finally, we move to properties that only hold for undercut′ and stable extensions. Let in the following $\mathcal{E}$ be a stable extension based on $\mathbb{K}$.

Lemma 4 (Aggregation for Undercut′ and Stable Semantics)**.** Let $a_1, a_2 \in \mathcal{E}$ and $\mathsf{Con}(a_1), \mathsf{Con}(a_2) \vdash_{\mathcal{C}} \phi$. Then $a_3 = \langle \mathsf{Sup}(a_1) \cup \mathsf{Sup}(a_2), \phi \rangle \in \mathcal{E}$.

PROOF. Let $a_1, a_2 \in \mathcal{E}$, $\mathsf{Con}(a_1), \mathsf{Con}(a_2) \vdash_{\mathcal{C}} \phi$ and $a_3 = \langle \mathsf{Sup}(a_1) \cup \mathsf{Sup}(a_2), \phi \rangle$. Suppose b undercuts′ [resp. h-undercuts] a_3. So, $\mathsf{Sup}(b) \vdash_{\mathcal{C}} \neg \bigwedge(\mathsf{Sup}(a_1) \cup \mathsf{Sup}(a_2))$ and hence $\mathsf{Sup}(b) \cup \mathsf{Sup}(a_1) \cup \mathsf{Sup}(a_2) \vdash_{\mathcal{C}} \bot$. By Proposition 15, $b \notin \mathcal{E}$. By the stability of $\mathcal{E}$, $\mathcal{E}$ undercuts′ b. So, a_3 is defended by $\mathcal{E}$ and by the completeness of $\mathcal{E}$, $a_3 \in \mathcal{E}$. □

Proposition 16 (Support closure for Undercut′ and stable semantics)**.** If $a_1, \ldots, a_n \in \mathcal{E}$ and $b \in \mathsf{Arg}(\mathbb{K}) \cup \mathsf{HArg}(\mathbb{AF})$ is such that $\mathsf{Sup}(b) \subseteq \bigcup_{i=1}^{n} \mathsf{Sup}(a_i)$, then $b \in \mathcal{E}$.

PROOF. We consider the case in which $b \in \mathsf{HArg}(\mathbb{K})$. The other case is a simple variant of the given proof. So, let $b = [b_1, \ldots, b_m] \in \mathsf{HArg}(\mathbb{AF})$ such that $\mathsf{Sup}(b) \subseteq \bigcup_{i=1}^{n} \mathsf{Sup}(a_i)$. By Corollary 1 it is sufficient to show that $b_i \in \mathcal{E}$ for all $i \in \{1, \ldots, m\}$. Without loss of generality consider b_1. Then, $\mathsf{Sup}(b_1) \subseteq \bigcup_{i=1}^{n} \mathsf{Sup}(a_i)$. By Lemma 4 and induction, $a_{n+1} = \langle \bigcup_{i=1}^{n} \mathsf{Sup}(a_i), \bigwedge_{i=1}^{n} \mathsf{Con}(a_i) \rangle \in \mathcal{E}$.

By Lemma 1, $\mathsf{str}(@(\mathsf{Sup}(b_1))) \geq \mathsf{str}(@(\mathsf{Sup}(a_{n+1})))$. Suppose c undercuts$'$ [resp. h-undercuts] b_1. So, $\mathsf{Con}(c) \vdash_{\mathcal{C}} \neg \bigwedge \mathsf{Sup}(b_1)$ and, in the case of undercut$'$, $\mathsf{str}(c) \geq \mathsf{str}(@(\mathsf{Sup}(b_1)))$. We have, $\mathsf{Con}(c) \vdash_{\mathcal{C}} \neg \bigwedge \mathsf{Sup}(a_{n+1})$ (since $\bigwedge \mathsf{Sup}(a_{i+1}) \vdash_{\mathcal{C}} \bigwedge \mathsf{Sup}(b_1)$). Also, in the case of undercut$'$, $\mathsf{str}(c) \geq \mathsf{str}(@(\mathsf{Sup}(a_{n+1})))$. Thus, c undercuts$'$ [resp. h-undercuts] a_{n+1}. Since $a_{n+1} \in \mathcal{E}$, $\mathcal{E}$ undercuts$'$ [resp. h-undercuts] c. Altogether this shows that $\mathcal{E}$ defends b_1. By the completeness of $\mathcal{E}$, $b_1 \in \mathcal{E}$. □

Proposition 17 (Logical Closure for Undercut$'$ and Stable Semantics)**.** If $a_1, \ldots, a_n \in \mathcal{E}$, $\langle \bigcup_{i=1}^{n} \mathsf{Sup}(a_i), \phi \rangle \in \mathcal{E}$, for any ϕ for which $\mathsf{Con}(a_1), \ldots, \mathsf{Con}(a_n) \vdash_{\mathcal{C}} \phi$.

PROOF. Let $a_1, \ldots, a_n \in \mathcal{E}$. By using Lemma 4 inductively, $b_1 = \langle \bigcup_{i=1}^{n} \mathsf{Sup}(a_i), \bigwedge_{i=1}^{n} \mathsf{Con}(a_i) \rangle \in \mathcal{E}$. By Proposition 9, $b_2 = \langle \mathsf{Sup}(b_1), \phi \rangle \in \mathcal{E}$ for any ϕ for which $\mathsf{Con}(b_1) \vdash_{\mathcal{C}} \phi$. This concludes our proof, since $\mathsf{Con}(b_1) \vdash_{\mathcal{C}} \phi$ holds iff $\mathsf{Con}(a_1), \ldots, \mathsf{Con}(a_n) \vdash_{\mathcal{C}} \phi$. □

C. VARIANTS FOR THE HYPER-ARGUMENTATION FRAMEWORK

We start with a basic fact about the relation between defeats and h-defeats which we will (silently) use in what follows.

Fact 6. Given a knowledge base $\mathbb{K}$, let $a \in \mathsf{Arg}(\mathbb{K})$ and $b \in \mathsf{Arg}(\mathbb{K}) \cup \mathsf{HArg}(\mathbb{K})$.

1. b defeats a iff b defeats $[a]$.
2. b h-defeats a iff b h-defeats $[a]$.
3. If a defeats b then $[a]$ h-defeats b.

C.1. Defending from h-defeats with defeats

In this section we show that if we also allow for defeats to defend from h-defeats, we obtain the same complete extensions as in the system from Section 3.3. For this we define (with the difference to Definition 12 in item 2 in italic):

Definition 13. Given a knowledge base $\mathbb{K}$, $\mathcal{E}$ *defends'* some argument $a \in \mathsf{Arg}(\mathbb{K}) \cup \mathsf{HArg}(\mathbb{K})$ iff

1. for every defeater b of a there is a $c \in \mathcal{E}$ that defeats b, and
2. for every h-defeater b of a there is a $c \in \mathcal{E}$ that *defeats or* h-defeats b.

We say that $\mathcal{E}$ is *admissible'* iff it is conflict-free and defends' every $a \in \mathcal{E}$. $\mathcal{E}$ is *complete'* iff it is admissible' and it contains every $a \in \mathsf{Arg}(\mathbb{K}) \cup \mathsf{HArg}(\mathbb{K})$ that it defends'.

Proposition 18. Given a knowledge base $\mathbb{K}$, let $\mathbb{AF} = \langle \langle \mathsf{Arg}(\mathbb{K}), \mathsf{Harg}(\mathbb{K}) \rangle, \langle \mathsf{def}, \mathsf{Hdef} \rangle \rangle$. We have, $\mathcal{E}$ is complete in $\mathbb{AF}$ iff it is complete' in $\mathbb{AF}$.

PROOF. Let $\mathcal{E}$ be complete. We first show that it is admissible'. Trivially, it is conflict-free. Suppose a defeats [resp. h-defeats] some $b \in \mathcal{E}$. Since $\mathcal{E}$ is admissible, there is a $c \in \mathcal{E}$ that defeats [resp. h-defeats] a. Therefore $\mathcal{E}$ defends' b. So $\mathcal{E}$ is admissible'.

We now show that $\mathcal{E}$ is complete'. Suppose $\mathcal{E}$ defends' some a. We show that $\mathcal{E}$ also defends a and so by the completeness of $\mathcal{E}$, $a \in \mathcal{E}$. Suppose some b defeats a. So, there is a $c \in \mathcal{E}$ that defeats b. Suppose some b h-defeats a. So, there is some $c \in \mathcal{E}$ that defeats or h-defeats b. Suppose c defeats b. So, $c \in \mathsf{Arg}(\mathbb{K})$ and by Corollary 1 $[c] \in \mathcal{E}$. Note that $[c]$ h-defeats b. So in both cases $\mathcal{E}$ defends' a.

Let now $\mathcal{E}$ be complete'. We first show that it is admissible. Trivially, it is conflict-free. Suppose some a defeats some $b \in \mathcal{E}$. By the admissibility' of $\mathcal{E}$ there is a $c \in \mathcal{E}$ that defeats a. Suppose some a h-defeats some $b \in \mathcal{E}$. By the admissibility' of $\mathcal{E}$ there is a $c \in \mathcal{E}$ that defeats or h-defeats a. In the second case $\mathcal{E}$ also defends b. In the former case $[c]$ h-defeats a. We therefore show ($\star$) that $[c] \in \mathcal{E}$ which implies that $\mathcal{E}$ defends a.

Ad ($\star$). Suppose some d defeats [resp. h-defeats] $[c]$. Then d also defeats [resp. h-defeats] c. In view of this, since $\mathcal{E}$ defends' c it also defends $[c]$. By the completeness' of $\mathcal{E}$, $[c] \in \mathcal{E}$.

We now show that $\mathcal{E}$ is complete. Suppose $\mathcal{E}$ defends a. We show that it also defends' a and thus, by the completeness' of $\mathcal{E}$, $a \in \mathcal{E}$. This shows that $\mathcal{E}$ is complete. Suppose b defeats [h-defeats] a. Thus, there is a $c \in \mathcal{E}$ that defeats [h-defeats] b. So, $\mathcal{E}$ also defends' a. □

C.2. Identifying simple hyper-arguments with their regular counterparts

In Section 3.3 we allow for hyper-arguments of the form $[a]$, which leads to the redundancy that a framework will contain for each argument a also the corresponding hyper-argument $[a]$. This can, however, easily be avoided, that is, we can identify regular arguments with their corresponding hyper-arguments. For this, we merely have to slightly adjust the definition of hyper-arguments and the definition of hyper-defeat.

1. We let Hyper-arguments (with capital "H") be of the form $[a_1, \ldots, a_n]$, where $n \geq 2$ and $a_1, \ldots, a_n$ are regular arguments. We denote the set of all Hyper-arguments for a knowledge base $\mathbb{K}$ by $\mathsf{HARG}(\mathbb{K})$. Note that $\mathsf{HArg}(\mathbb{K}) \supseteq \mathsf{HARG}(\mathbb{K})$.
2. We enhance the definition of h-defeats (see Definition 9) to the domain $(\mathsf{Arg}(\mathbb{K}) \cup \mathsf{HARG}(\mathbb{K})) \times (\mathsf{Arg}(\mathbb{K}) \cup \mathsf{HARG}(\mathbb{K}))$, resulting in H-defeats (with capital "H"), as follows. On the domain $\mathsf{HARG}(\mathbb{K}) \times (\mathsf{Arg}(\mathbb{K}) \cup \mathsf{HARG}(\mathbb{K}))$ we let H-defeats be defined as h-defeats in Definition 9. Moreover, we let $a \in \mathsf{Arg}(\mathbb{K})$ H-defeat some $b \in \mathsf{Arg}(\mathbb{K}) \cup \mathsf{HARG}(\mathbb{K})$ iff $[a]$ h-defeats b.

Proposition 19. Given a knowledge base $\mathbb{K}$, let $\mathbb{AF} = \langle \langle \mathsf{Arg}(\mathbb{K}), \mathsf{HArg}(\mathbb{K}) \rangle, \langle \mathsf{Def}, \mathsf{Hdef} \rangle \rangle$ and $\mathbb{AF}_H = \langle \langle \mathsf{Arg}(\mathbb{K}), \mathsf{HARG}(\mathbb{K}) \rangle, \langle \mathsf{Def}, \mathsf{HDEF} \rangle \rangle$, where HDef is the regular notion of h-defeat and HDEF is H-defeat.

1. If $\mathcal{E}$ is complete in $\mathbb{AF}$ then $\mathcal{E}' = \mathcal{E} \cap (\mathsf{Arg}(\mathbb{K}) \cup \mathsf{HARG}(\mathbb{K}))$ is complete in $\mathbb{AF}_H$.
2. If $\mathcal{E}'$ is complete in $\mathbb{AF}_H$ then $\mathcal{E} = \mathcal{E}' \cup \{[a] \mid a \in \mathcal{E}' \cap \mathsf{Arg}(\mathbb{K})\}$ is complete in $\mathbb{AF}$.

PROOF. *Ad 1.* Let $\mathcal{E}$ be a complete extension of $\mathbb{AF}$ and let $\mathcal{E}'$ be as in item 1.

Conflict-freeness. Assume for a contradiction that $\mathcal{E}'$ is not conflict-free in $\mathbb{AF}_H$. By the conflict-freeness of $\mathcal{E}$ and since $\mathcal{E}' \subseteq \mathcal{E}$, there is a $a \in \mathsf{Arg}(\mathbb{K}) \cap \mathcal{E}$ that H-defeats some argument $b \in \mathcal{E}'$. So, $[a]$ h-defeats b. By Corollary 1, $[a] \in \mathcal{E}$. Since also $b \in \mathcal{E}$ this contradicts the conflict-freeness of $\mathcal{E}$.

Admissibility. Suppose first some $a \in \mathsf{Arg}(\mathbb{K})$ defeats some $b \in \mathcal{E}'$. Since $b \in \mathcal{E}$ and by the admissibility of $\mathcal{E}$ there is a $c \in \mathcal{E}$ that defeats a. Note that $c \in \mathsf{Arg}(\mathbb{K})$ and so $c \in \mathcal{E}'$.

Suppose now that some $a = [a_1, \ldots, a_n] \in \mathsf{HARG}(\mathbb{K})$ H-defeats some $b \in \mathcal{E}'$. Since $a \in \mathsf{HArg}(\mathbb{K})$, it also h-defeats b. Since $b \in \mathcal{E}$ and by the admissibility of $\mathcal{E}$, there is a $[c_1, \ldots, c_m] \in \mathcal{E}$ that h-defeats a. If $m \geq 2$, $[c_1, \ldots, c_m] \in \mathcal{E}'$ H-defeats a. Else, $c_1 \in \mathcal{E}$ by Corollary 1 and c_1 H-defeats a.

Finally suppose some $a \in \mathsf{Arg}(\mathbb{K})$ H-defeats some $b \in \mathcal{E}'$. Then $[a]$ h-defeats b. By the admissibility of $\mathcal{E}$ and since $b \in \mathcal{E}$, there is a $[c_1, \ldots, c_m] \in \mathcal{E}$ that h-defeats $[a]$. If $m \geq 2$, $[c_1, \ldots, c_m] \in \mathcal{E}'$ H-defeats a. Else, $c_1 \in \mathcal{E}$ by Corollary 1 and c_1 H-defeats a. So, $\mathcal{E}'$ is admissible in $\mathbb{AF}_H$.

Completeness. Suppose $\mathcal{E}'$ defends some $b \in \mathsf{Arg}(\mathbb{K}) \cup \mathsf{HARG}(\mathbb{K})$ in $\mathbb{AF}_H$. We show that $\mathcal{E}$ also defends b in $\mathbb{AF}$ and, by the completeness of $\mathcal{E}$, $b \in \mathcal{E}$ and therefore $b \in \mathcal{E}'$. Suppose some $a \in \mathsf{Arg}(\mathbb{K}) \cup \mathsf{HArg}(\mathbb{K})$ defeats or h-defeats b.

- Suppose first that a defeats b. So, $a \in \mathsf{Arg}(\mathbb{K})$ and since $\mathcal{E}'$ defends b there is a $c \in \mathsf{Arg}(\mathbb{K}) \cap \mathcal{E}'$ that defeats a. Note that $c \in \mathcal{E}$.
- Suppose now that a h-defeats b. Then $a \in \mathsf{HArg}(\mathbb{K})$. If $a \in \mathsf{HARG}(\mathbb{K})$, a also H-defeats b and so there is a $c \in \mathcal{E}'$ that H-defeats a. If $c \in \mathsf{Arg}(\mathbb{K})$, $c \in \mathcal{E}$ and $[c] \in \mathcal{E}$ by Corollary 1. Also $[c]$ h-defeats a. Else $c \in \mathsf{HARG}(\mathbb{K})$ and so $c \in \mathsf{HArg}(\mathbb{K}) \cap \mathcal{E}$ h-defeats b.

Altogether this shows that $\mathcal{E}$ defends b and so $b \in \mathcal{E}$. Hence, $b \in \mathcal{E}'$.

Ad 2. Let $\mathcal{E}'$ be a complete extension of $\mathbb{AF}_H$ and let $\mathcal{E}$ be as in item 2.

Conflict-freeness. Assume for a contradiction that $\mathcal{E}$ is not conflict-free in $\mathbb{AF}$. So, there are conflicting $a, b \in \mathcal{E}$ and a defeats or h-defeats b. In the former case and by the conflict-freeness of $\mathcal{E}'$ in $\mathbb{AF}_H$, $b = [b'] \notin \mathcal{E}'$. But then a H-defeats b' and $b' \in \mathcal{E}'$, in contradiction to the conflict-freeness of $\mathcal{E}'$. Else, if a h-defeats b, either $a = [a'] \notin \mathcal{E}'$ and $b \in \mathcal{E}'$, or $b = [b'] \notin \mathcal{E}'$ and $a \in \mathcal{E}'$, or $a = [a'] \notin \mathcal{E}'$ and $b = [b'] \notin \mathcal{E}'$. Then $a' \in \mathcal{E}'$ H-defeats b or a H-defeats $b' \in \mathcal{E}'$ or $a' \in \mathcal{E}'$ H-defeats $b' \in \mathcal{E}'$. In each case we reach a contradiction with the conflict-freeness of $\mathcal{E}'$ in $\mathbb{AF}_H$.

Admissibility. Suppose first that some $a \in \mathsf{Arg}(\mathbb{K})$ defeats some $b \in \mathcal{E}$. If $b \in \mathcal{E}'$ and by the admissibility of $\mathcal{E}'$ there is a $c \in \mathcal{E}'$ that defeats a. Note that $c \in \mathcal{E} \cap \mathsf{Arg}(\mathbb{K})$. Else, b is of the form $[b'] \in \mathsf{HArg}(\mathbb{K}) \setminus \mathsf{HARG}(\mathbb{K})$. So, $b' \in \mathcal{E}'$ and a defeats b'. By the admissibility of $\mathcal{E}'$ there is a $c \in \mathcal{E}' \cap \mathsf{Arg}(\mathbb{K})$ that defeats a. Note that $c \in \mathcal{E}$.

Suppose now that $a = [a_1, \ldots, a_m] \in \mathsf{HArg}(\mathbb{K})$ h-defeats some $b \in \mathcal{E}$.

- If $b \in \mathsf{Arg}(\mathbb{K}) \cup \mathsf{HARG}(\mathbb{K})$ then $b \in \mathcal{E}'$ and so, by the admissibility of $\mathcal{E}'$ in $\mathbb{AF}_H$, there is a $c \in \mathcal{E}'$ that H-defeats b.
- Else, if $b = [b'] \in \mathsf{HArg}(\mathbb{K}) \setminus \mathsf{HARG}(\mathbb{K})$, then $b' \in \mathcal{E}'$ and a H-defeats b'. Therefore, there is a $c \in \mathcal{E}'$ that H-defeats a.

In both cases, if $c \notin \mathsf{Arg}(\mathbb{K})$, $c \in \mathsf{HArg}(\mathbb{K})$ and so $c \in \mathcal{E}$ also h-defeats b. Else, $[c] \in \mathcal{E}$. Note that $[c]$ h-defeats b.

Completeness. Suppose $\mathcal{E}$ defends some $b \in \mathsf{Arg}(\mathbb{K}) \cup \mathsf{HArg}(\mathbb{K})$ in $\mathbb{AF}$. We have to show that $b \in \mathcal{E}$.

(1) Suppose first that $b = [b'] \in \mathsf{HArg}(\mathbb{K}) \setminus \mathsf{HARG}(\mathbb{K})$. We show that $b' \in \mathcal{E}'$ by showing that $\mathcal{E}'$ defends b' (and in view of the completeness of $\mathcal{E}'$). In that case also $b \in \mathcal{E}$ by the definition of $\mathcal{E}$ in item 2. So, suppose some $a \in \mathsf{Arg}(\mathbb{K}) \cup \mathsf{HARG}(\mathbb{K})$ defeats or H-defeats b'.

- In the case of defeat a also defeats b and so there is a $c \in \mathcal{E}$ that defeats a. Since $c \in \mathsf{Arg}(\mathbb{K})$, $c \in \mathcal{E}'$.
- Consider the case of H-defeat. Suppose first that $a \in \mathsf{Arg}(\mathbb{K})$. So, $[a]$ h-defeats b. Since $\mathcal{E}$ defends b there is a $c \in \mathcal{E}$ that h-defeats $[a]$. If $c = [c'] \notin \mathcal{E}'$, $c' \in \mathcal{E}'$ H-defeats a. Else $c \in \mathsf{HARG}(\mathbb{K}) \cap \mathcal{E}'$ H-defeats a.

  Suppose $a \in \mathsf{HARG}(\mathbb{K})$. Since $\mathcal{E}$ defends b there is a $c \in \mathsf{HArg}(\mathbb{K}) \cap \mathcal{E}$ that h-defeats a. If $c = [c'] \notin \mathsf{HARG}(\mathbb{K})$, $c' \in \mathcal{E}'$ H-defeats a. Else, $c \in \mathsf{HARG}(\mathbb{K}) \cap \mathcal{E}'$ H-defeats a.

Altogether we have shown that $\mathcal{E}'$ defends b' and so $b' \in \mathcal{E}'$ by the completeness of $\mathcal{E}'$. So $b \in \mathcal{E}$.

(2) Suppose now that $b \in \mathsf{Arg}(\mathbb{K}) \cup \mathsf{HARG}(\mathbb{K})$. We show that $\mathcal{E}'$ defends b and so by the completeness of $\mathcal{E}'$, $b \in \mathcal{E}'$ and therefore $b \in \mathcal{E}$. So, suppose some $a \in \mathsf{Arg}(\mathbb{K}) \cup \mathsf{HARG}(\mathbb{K})$ defeats or H-defeats b.

- In case of defeat, there is a $c \in \mathsf{Arg}(\mathbb{K}) \cap \mathcal{E}$ that defeats a. So, $c \in \mathcal{E}'$.
- Consider the case of H-defeat. Suppose first that $a \in \mathsf{Arg}(\mathbb{K})$. So, $[a]$ h-defeats b. Since $\mathcal{E}$ defends b there is a $c \in \mathcal{E}$ that h-defeats $[a]$. If $c = [c'] \notin \mathcal{E}'$, $c' \in \mathcal{E}'$ H-defeats a. Else $c \in \mathsf{HARG}(\mathbb{K}) \cap \mathcal{E}'$ H-defeats a.

  Suppose $a \in \mathsf{HARG}(\mathbb{K})$. Since $\mathcal{E}$ defends b there is a $c \in \mathsf{HArg}(\mathbb{K}) \cap \mathcal{E}$ that h-defeats a. If $c = [c'] \notin \mathsf{HARG}(\mathbb{K})$, $c' \in \mathcal{E}'$ H-defeats a. Else, $c \in \mathsf{HARG}(\mathbb{K}) \cap \mathcal{E}'$ H-defeats a.

Altogether we have shown that $\mathcal{E}'$ defends b and so $b \in \mathcal{E}'$ by the completeness of $\mathcal{E}'$. □

The following result follows immediately with Propositions 18 and 19.

Corollary 2. Given a knowledge base $\mathbb{K}$, let $\mathbb{AF} = \langle\langle \mathsf{Arg}(\mathbb{K}), \mathsf{Harg}(\mathbb{K})\rangle, \langle \mathsf{def}, \mathsf{Hdef}\rangle\rangle$ and $\mathbb{AF}_H = \langle\langle \mathsf{Arg}(\mathbb{K}), \mathsf{HARG}(\mathbb{K})\rangle, \langle \mathsf{def}, \mathsf{Hdef}\rangle\rangle$. We have:

1. If $\mathcal{E}$ is complete' in $\mathbb{AF}$ then $\mathcal{E}' = \mathcal{E} \setminus \{[a] \mid a \in \mathsf{Arg}(\mathbb{K}) \cap \mathcal{E}\}$ is complete in $\mathbb{AF}_H$.
2. If $\mathcal{E}'$ is complete in $\mathbb{AF}_H$ then $\mathcal{E} = \mathcal{E}' \cup \{[a] \mid a \in \mathsf{Arg}(\mathbb{K}) \cap \mathcal{E}'\}$ is complete' in $\mathbb{AF}$.

D. THE QUESTIONAIRE

The questionnaire presents three scenarios, each with its own questions. The questions concern the estimation of the strength of a given argument in a scale from "very weak" to "very strong" (each with 10 subdivisions, see Figure 5). We now present the three scenarios.

Scenario (S1. An urn of balls**).** Consider an urn with 90 single-colored balls. 30 of them are red while 60 are either black or yellow. You do not know the distribution of the yellow and the black balls. The balls are mixed up well. You draw one ball out of the urn.

Consider the following:

Bet 1 You bet on drawing a red ball.
Bet 2 You bet on drawing a black ball.
Bet 3 You bet on drawing a red or yellow ball. [*This means that you win when a red or yellow ball is drawn in the single draw. Analogously for the following questions.*]
Bet 4 You bet on drawing a black or yellow ball.
Bet 5 You bet on drawing a black or yellow or red ball.

Please answer the following questions.

> **Q1** How strong is an argument for choosing bet 1?
> **Q2** How strong is an argument for choosing bet 2?
> **Q3** How strong is an argument for choosing bet 3?
> **Q4** How strong is an argument for choosing bet 4?
> **Q5** How strong is an argument for choosing bet 5?

Scenario (S2.1. Medical Diagnosis**).** A patient comes in with exactly one symptom. The symptom leads to

- disease A or B in $2/3$ of cases (but you don't know in which distribution), and

state	r	y	b	P_λ	argument	dsp	dps	mean	bst$_m$	precMean	qu.
s_1	1	0	0	$1/3$	$a_1 : \langle\{r\}, \texttt{stra}_1\rangle$	$1/3$	$1/3$	$1/3$	$1/3$	$1/3$	Q1
s_2	0	1	0	λ	$a_2 : \langle\{b\}, \texttt{stra}_2\rangle$	0	$2/3$	$1/3$	$2/3m$	$1/9$	Q2
s_3	0	0	1	$2/3 - \lambda$	$a_3 : \langle\{r \vee y\}, \texttt{stra}_3\rangle$	$1/3$	1	$2/3$	$(m+2)/3m$	$2/9$	Q3
s_4	0	1	1	0	$a_4 : \langle\{b \vee y\}, \texttt{stra}_4\rangle$	$2/3$	$2/3$	$2/3$	$2/3$	$2/3$	Q4
$\vdots$	$\vdots$	$\vdots$	$\vdots$	$\vdots$	$a_5 : \langle\{b \vee y \vee r\}, \texttt{stra}_5\rangle$	1	1	1	1	1	Q5

Table S1. The formal model of S1, Example 21.

- disease C in $1/3$ of cases.

A person can only have one of the three diseases (A, B, or C) at a time. Suppose you are a doctor and this is the only information you have at hand. Given this information, how strong is an argument for the claim …

Q8 …that the patient has disease A or B?
Q9 …that the patient has disease C?
Q10 …that the patient has disease C or B?
Q11 …that the patient has disease B?
Q12 …that the patient has disease A or C?
Q13 …that the patient has disease A?
Q14 …that the patient has disease A or B or C?

Scenario (S2.2. Medical diagnosis: more information**).** You are facing the same scenario as before, but you have one additional information (marked with a $\star$ below): A patient comes in with exactly one symptom. The symptom leads to

- disease A or B in $2/3$ of cases (but you don't know in which distribution),
- disease C in $1/3$ of cases, and

$\star$ disease B is more common than disease A.

A person can only have one of the three diseases (A, B, or C) at a time. Suppose you are a doctor and this is the only information you have at hand. Given this information, how strong is an argument for the claim …

Q15 …that the patient has disease C?
Q16 …that the patient has disease B?
Q17 …that the patient has disease A?

E. MODELING THE EXAMPLES FROM THE QUESTIONNAIRE

Example 21 (Ellsberg, S1, Questions 1–5)**.** We model the Ellberg scenario in terms of the knowledge base $\mathbb{K} = \langle\langle \mathcal{V}_p : \{r, y, b\}, \mathcal{V}_l : \{\texttt{stra}_i \mid i = 1, \dots, 5\}\rangle, \mathcal{A} : \mathsf{sent}(\mathcal{V}_p), \mathcal{C}, \mathbb{P} : \{P_\lambda \mid 0 \leq \lambda \leq 2/3\}\rangle$ where $\mathcal{C} = \{\neg r \leftrightarrow (y \vee b), \neg(y \wedge b), r \leftrightarrow \texttt{stra}_1, b \leftrightarrow \texttt{stra}_2, (r \vee y) \leftrightarrow \texttt{stra}_3, (b \vee y) \leftrightarrow \texttt{stra}_4, (b \vee y \vee r) \leftrightarrow \texttt{stra}_5\}$ and P_λ is as in Table S1.

Example 22 (Ellsberg, S1, alternative model, Questions 1–5)**.** Alternatively, we can model Ellsberg in terms of the knowledge base $\mathbb{K} = \langle\langle \mathcal{V}_p : \{r\}, \mathcal{V}_l : \{b, y, \texttt{stra}_i \mid i = 1, \dots, 5\}\rangle, \mathcal{A} : \{r, \neg r, r \vee \neg r\}, \mathcal{C}, \{P\}\rangle$ where $\mathcal{C} = \{\neg r \leftrightarrow (y \vee b), \neg(y \wedge b), r \leftrightarrow \texttt{stra}_1, b \leftrightarrow \texttt{stra}_2, (r \vee y) \leftrightarrow \texttt{stra}_3, (b \vee y) \leftrightarrow \texttt{stra}_4, (b \vee y \vee r) \leftrightarrow \texttt{stra}_5\}$ and P as in Table S2. Our arguments are now: $a_1 : \langle\{r\}, \texttt{stra}_1\rangle$, $a_2 : \langle\{\neg r\}, \texttt{stra}_2\rangle$,

state	r	P_λ	y	b	$\mathtt{stra}_1$	$\mathtt{stra}_2$	$\mathtt{stra}_3$	$\mathtt{stra}_4$	$\mathtt{stra}_5$
s_1	0	$2/3$	$\lozenge$	$\lozenge$	0	$\lozenge$	$\lozenge$	1	1
s_2	1	$1/3$	0	0	1	0	1	0	1

Table S2. Alternative formal model of S1, Example 22.

	a	b	c	P_λ	P_μ
s_1	1	0	0	λ	μ
s_2	0	1	0	$2/3-\lambda$	$1/3+\mu$
s_3	0	0	1	$1/3$	$1/3$
s_4	0	1	1	0	0
$\vdots$	$\vdots$	$\vdots$	$\vdots$	$\vdots$	$\vdots$

argument	dsp	dps	mean	bst_m	precMean	
$a_8 : \langle\{a \vee b\}, a \vee b\rangle$	$2/3$	$2/3$	$2/3$	$2/3$	$2/3$	Q8
$a_9 : \langle\{c\}, c\rangle$	$1/3$	$1/3$	$1/3$	$1/3$	$1/3$	Q9
$a_{10} : \langle\{b \vee c\}, b \vee c\rangle$	$1/3$	1	$2/3$	$(m+2)/3m$	$2/9$	Q10
$a_{11} : \langle\{b\}, b\rangle$	0	$2/3$	$1/3$	$2/3m$	$1/9$	Q11
$a_{12} : \langle\{a \vee c\}, a \vee c\rangle$	$1/3$	1	$2/3$	$(m+2)/3m$	$2/9$	Q12
$a_{13} : \langle\{a\}, a\rangle$	0	$2/3$	$1/3$	$2/3m$	$1/9$	Q13
$a_{14} : \langle\{a \vee b \vee c\}, a \vee b \vee c\rangle$	1	1	1	1	1	Q14
$a_{15} : \langle\{c\}, c\rangle$	$1/3$	$1/3$	$1/3$	$1/3$	$1/3$	Q15
$a_{16} : \langle\{b\}, b\rangle$	$1/3$	$2/3$	$1/2$	$(m+1)/3m$	$1/3$	Q16
$a_{17} : \langle\{a\}, a\rangle$	0	$1/3$	$1/6$	$1/3m$	$1/9$	Q17

Table S3. The formal model of S2, Example 23.

$a_3 : \langle\{r\}, \mathtt{stra}_3\rangle$, $a_4 : \langle\{\neg r\}, \mathtt{stra}_4\rangle$ and $a_5 : \langle\{r \vee \neg r\}, \mathtt{stra}_5\rangle$. They have exactly the same strengths as the respective arguments in Table S1.

Example 23 (Medical diagnosis, S2, Questions 8–17)**.** We model the medical scenario of Example 2 in the questionnaire in terms of the knowledge base $\mathbb{K} = \langle\langle\mathcal{V}_p : \{a, b, c\}, \mathcal{V}_l : \emptyset\rangle, \mathcal{A} : \mathsf{sent}(\mathcal{V}_p), \mathcal{C}, \mathbb{P} : \{P_\lambda \mid \lambda \in [0, 2/3]\}\rangle$ as in Table S3. The variant from Example 2′ is given by $\mathbb{P}' = \{P_\mu \mid \mu \in [0, 1/3]\}$.
